# Angiotensin IV attenuates diabetic cardiomyopathy *via* suppressing FoxO1-induced excessive autophagy, apoptosis and fibrosis

**DOI:** 10.7150/thno.48561

**Published:** 2021-07-25

**Authors:** Meng Zhang, Wenhai Sui, Yanqiu Xing, Jing Cheng, Cheng Cheng, Fei Xue, Jie Zhang, Xiaohong Wang, Cheng Zhang, Panpan Hao, Yun Zhang

**Affiliations:** 1 The Key Laboratory of Cardiovascular Remodeling and Function Research, Chinese Ministry of Education, Chinese National Health Commission and Chinese Academy of Medical Sciences, The State and Shandong Province Joint Key Laboratory of Translational Cardiovascular Medicine, Department of Cardiology, Qilu Hospital, Cheeloo College of Medicine, Shandong University, Jinan 250012, Shandong, China.; 2 Department of Geriatrics, Qilu Hospital, Cheeloo College of Medicine, Shandong University, Key Laboratory of Cardiovascular Proteomics of Shandong Province, Jinan 250012, Shandong, China.; 3 Beijing Chaoyang Hospital, Capital Medical University, Beijing 100020, China.

**Keywords:** angiotensin IV, diabetic cardiomyopathy, autophagy, FoxO1

## Abstract

**Rationale:** The rennin-angiotensin-aldosterone system (RAAS) plays a critical role in the pathogenesis of diabetic cardiomyopathy, but the role of a member of RAAS, angiotensin IV (Ang IV), in this disease and its underlying mechanism are unclear. This study was aimed to clarify the effects of Ang IV and its downstream mediator forkhead box protein O1 (FoxO1) on diabetic cardiomyopathy.

**Methods:***In vivo*, diabetic mice were treated with low-, medium- and high-dose Ang IV, AT_4_R antagonist divalinal, FoxO1 inhibitor AS1842856 (AS), or their combinations. *In vitro*, H9C2 cardiomyocytes and cardiac fibroblasts were treated with different concentrations of glucose, low-, medium- and high-dose Ang IV, divalinal, FoxO1-overexpression plasmid (FoxO1-OE), AS, or their combinations.

**Results:** Ang IV treatment dose-dependently attenuated left ventricular dysfunction, fibrosis, and myocyte apoptosis in diabetic mice. Besides, enhanced autophagy and FoxO1 protein expression by diabetes were dose-dependently suppressed by Ang IV treatment. However, these cardioprotective effects of Ang IV were completely abolished by divalinal administration. Bioinformatics analysis revealed that the differentially expressed genes were enriched in autophagy, apoptosis, and FoxO signaling pathways among control, diabetes, and diabetes+high-dose Ang IV groups. Similar to Ang IV, AS treatment ameliorated diabetic cardiomyopathy in mice. *In vitro*, high glucose stimulation increased collagen expression, apoptosis, overactive autophagy flux and FoxO1 nuclear translocation in cardiomyocytes, and upregulated collagen and FoxO1 expression in cardiac fibroblasts, which were substantially attenuated by Ang IV treatment. However, these protective effects of Ang IV were completely blocked by the use of divalinal or FoxO1-OE, and these detrimental effects were reversed by the additional administration of AS.

**Conclusions:** Ang IV treatment dose-dependently attenuated left ventricular dysfunction and remodeling in a mouse model of diabetic cardiomyopathy, and the mechanisms involved stimulation of AT_4_R and suppression of FoxO1-mediated fibrosis, apoptosis, and overactive autophagy.

## Introduction

The prevalence of both type 1 and type 2 diabetes mellitus (DM) is increasing rapidly worldwide and DM has been recognized as an independent risk factor for heart failure 1, 2. The pathogenesis of diabetic cardiomyopathy involves activated rennin-angiotensin-aldosterone system (RAAS), oxidative stress, lipotoxicity, maladaptive immune responses, imbalanced mitochondrial dynamics, impaired myocyte autophagy, increased myocyte apoptosis and fibrosis, which ultimately lead to left ventricular diastolic and systolic dysfunction 3-7. Of paramount importance is the activation of the circulatory and myocardial RAAS by hyperglycemia 4, 6. Our previous studies demonstrated that angiotensin-(1-7) [Ang-(1-7)], which is cleaved from Ang II by angiotensin-converting enzyme 2 (ACE2), counteracts the deleterious effects of the ACE-Ang II-Ang II type 1 receptor (AT_1_R) axis, and thus, the ACE2-Ang-(1-7)-Mas receptor (MasR) axis has been recognized as a promising approach to the treatment of cardiovascular diseases including diabetic cardiomyopathy 8-10. However, the relationship between diabetic cardiomyopathy and a new member of RAAS, angiotensin IV (Ang IV) peptide, has not yet been reported in the literature.

Ang IV, also known as Ang-(3-8), is hydrolyzed from Ang II by dipeptidyl aminopeptidase III (DPP3) or from Ang III by aminopeptidase N (APN) 11. This hexapeptide Ang IV was initially considered biologically inactive because it has a very weak affinity with AT_1_R and AT_2_R, but subsequently Ang IV was found effective for correcting memory deficits in animals with amnesia *via* stimulation of AT_4_R, a newly discovered receptor specifically binding to Ang IV with a high-affinity. Although mainly concentrated in the brain, AT_4_R is distributed in a wide range of tissues including heart, blood vessels, kidney, adrenal gland and lungs 12. Recently, the Ang IV-AT_4_R axis has been recognized to play an important role in antagonizing the effect of Ang II. In rat hearts, treatment with Ang IV improved myocardial injury induced by Ang II and ischemia/reperfusion 13, 14. Studies *in vitro* showed that Ang IV inhibited Ang II-induced apoptosis and hypertrophy of cardiomyocytes, as well as proliferation and collagen synthesis of cardiac fibroblasts 13. These preliminary findings suggest that the Ang IV-AT_4_R axis may protect against Ang II-induced myocardial injury. Nonetheless, the role of this novel axis in diabetic cardiomyopathy remains elusive.

Constitutive autophagy facilitates the elimination of dysfunctional organelles *via* selective and non-selective targeting approaches, which helps to maintain cellular homeostasis in the heart 15, 16. However, autophagic activity is disturbed in the heart under various stress states including starvation, diabetes, hypertension and ischemia, and an unrestrained autophagic activity accentuates the maladaptive cardiac remodeling in response to stress and contributes to the development of cardiomyopathy 15, 16. However, the precise role of autophagy in diabetic cardiomyopathy and its underlying mechanism remain obscure. Ang II was found to promote the production of autophagosomes in neonatal cardiomyocytes mediated *via* AT_1_R, which was constitutively antagonized by co-expression of AT_2_R 17. Additionally, Ang II is known to promote oxidative stress, myocyte hypertrophy, autophagy, and fibrosis in the heart, whereas Ang-(1-7) was able to antagonize these detrimental effects *via* stimulation of MasR 18. Thus, the Ang-(1-7)-MasR/AT_2_R axis is involved in the regulation of cardiomyocyte autophagy. In rat ventricles with ischemia-reperfusion injury, pretreatment with Ang IV suppressed apoptosis *via* phosphorylation of Akt and mammalian target of rapamycin (mTOR), two important negative regulators of autophagy 14. However, little is known about the role and mechanism of the Ang IV-AT_4_R axis in modulating autophagy in diabetic cardiomyopathy.

Available evidence has demonstrated that forkhead box protein O (FoxO) isoform is abundantly expressed in the heart and is deemed as a critical metabolic regulator, particularly in diabetic cardiomyopathy characterized by metabolic disturbances 19. In addition, high glucose (HG) stimulation and oxidative stress may activate FoxO1, which regulates apoptosis- and autophagy-associated genes 20. Thus, we hypothesized that Ang IV protects against diabetic cardiomyopathy *via* stimulation of AT_4_R and inhibition of FoxO1-enhanced autophagy, and a series of *in vivo* and *in vitro* experiments were designed and performed in this study to test this hypothesis.

## Materials and Methods

See supplemental data for details.

### Ethics statement

All animal experiments were performed in accordance with the recommendations in the Guide for the Care and Use of Laboratory Animals published by the US National Institutes of Health (NIH publication no. 85-23 revised 1996) and approved by the Ethics Committee and the Scientific Investigation Board of Shandong University Qilu Hospital (No. DWLL-2017-016, Jinan, Shandong, China).

### Animal experiment

**Figure 1:**
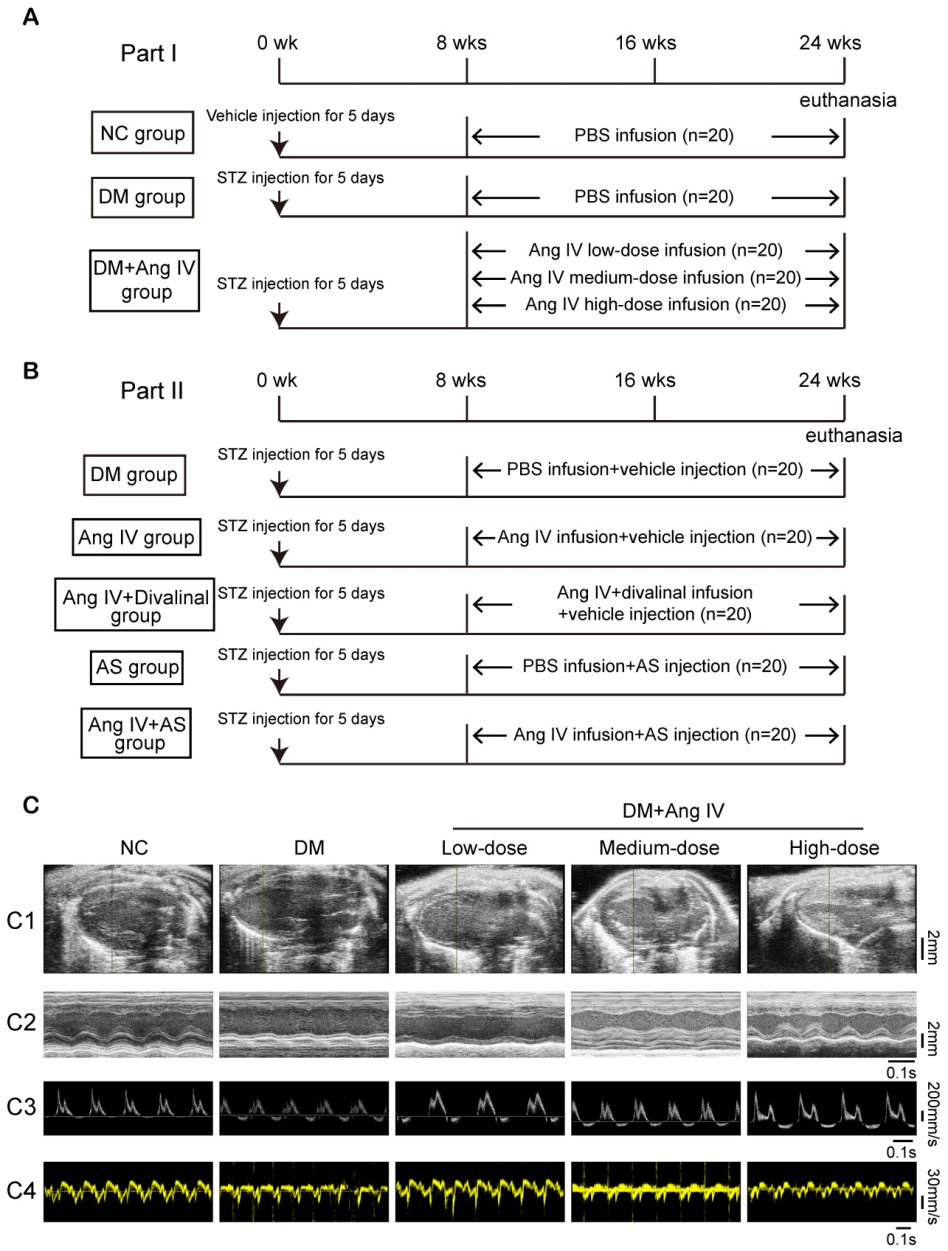
** The protocol of *in vivo* experiments and effect of Ang IV on left ventricular function in 5 groups of mice. (A)** Animal grouping and timeline of the first part of the *in vivo* experiment. **(B)** Animal grouping and timeline of the second part of the* in vivo* experiment.** (C)** Representative echocardiographic images in 5 groups of mice.** (C1)** Two-dimensional echocardiograms showing left ventricular long-axis views, scale bar in mm on the right; **(C2)** M-mode echocardiograms showing left ventricular dimensions and scale bar in mm on the right, and time stamp in seconds at the bottom; **(C3)** Pulse-wave Doppler echocardiograms depicting mitral inflow velocities, scale bar in mm/s on the right, and time stamp in seconds at the bottom; **(C4)** Tissue Doppler echocardiograms displaying mitral annular velocities, scale bar in mm/s on the right, and time stamp in seconds at the bottom. Ang IV: angiotensin IV; AS: FoxO1 inhibitor AS1842856; DM: diabetes mellitus; NC: normal control; STZ: streptozotocin; wks: weeks.

Animal experiments in the present study were divided into two parts. In the first part, to examine the dose-effect of Ang IV on diabetic cardiomyopathy, 100 eight-week-old male mice with C57BL/6J background were fed with normal chow diet and randomly separated into 5 groups (n=20 each): normal control (NC), DM, DM+low-dose Ang IV, DM+medium-dose Ang IV and DM+high-dose Ang IV, respectively. Diabetes was induced in mice *via* intraperitoneal injection of streptozotocin (STZ, Sigma, St Louis, MO; 55 mg/kg daily; dissolved in 0.1 mL of citrate buffer, pH 4.5) for 5 days consecutively. Two weeks after STZ injection, mice with fasting blood glucose (FBG) ≥16.7 mM were chosen for subsequent experiments. In the NC group, mice were intraperitoneally injected with vehicle (0.1 mL of citrate buffer, pH 4.5) daily for 5 days. Low-, medium-, or high-dose Ang IV (0.72 mg/kg/day, 1.44 mg/kg/day, and 2.88 mg/kg/day, respectively; HY-P1515A, MCE, NJ) was infused in the three treatment groups of mice from the end of 8 weeks to the end of 24 weeks. All groups were infused with normal saline or drugs *via* a subcutaneous osmotic mini-pump (Alza, Palo Alto, CA) (Figure 1A). The pump was implanted when deep reflexes disappeared after anesthesia using 2% isoflurane insufflation. Mice were kept in warm conditions until full recovery from anesthesia.

In the second part, in an attempt to elaborate the roles of AT_4_R and FoxO1 in the effect of Ang IV on diabetic cardiomyopathy, diabetes was induced in 100 mice using the method described above, which were randomly divided into 5 groups (n=20 per group): DM group that received an infusion of vehicle alone, Ang IV group that received an infusion of Ang IV of 2.88 mg/kg/day because high-dose Ang IV showed the best efficacy in the first part of the experiment, Ang IV+divalinal group that received an infusion of Ang IV plus AT_4_R antagonist divalinal (2.88 mg/kg/day, Lintai Biological Technology Company, Xi'an, China), AS group that received an infusion of FoxO1 inhibitor AS1842856 (AS, 20 mg/kg/day, HY-100596, MCE), and Ang IV+AS group that received an infusion of Ang IV plus FoxO1 inhibitor. Ang IV and divalinal-Ang IV were subcutaneously administered *via* an osmotic mini-pump as described above and AS was intraperitoneally injected for 16 weeks. Euthanasia was performed by cervical dislocation when deep reflexes disappeared under 2% isoflurane anesthesia. All mice underwent euthanasia for further pathological studies (Figure 1B).

### Cell culture

H9C2 cardiomyocytes (CRL-1446; ATCC, Gaithersburg, MD) and primary cardiac fibroblasts were cultured in Dulbecco's modified Eagle's medium (DMEM; Gibco BRL, Gaithersburg) containing 5% fetal bovine serum at 37 °C with 5% CO_2_. In the first part of the* in vitro* experiment, cardiomyocytes were cultured with different concentrations of glucose (5.5, 15, 25, 33.3, and 60 mM, respectively) for 24 h. In the second part of the *in vitro* experiment, 5.5 mM glucose (normal glucose control, Con group); 5.5 mM glucose plus 54.5 mM mannitol (high osmotic control, HO group), 60 mM glucose (high glucose, HG), and 60 mM glucose plus 10^-9^ M Ang IV, 60 mM glucose plus 10^-7^ M Ang IV and 60 mM glucose plus 10^-5^ M Ang IV were respectively added to cardiomyocytes and cardiac fibroblasts 24 h before harvest. In the third part of the *in vitro* experiment, vehicle, Ang IV (10^-5^ M), Ang IV plus divalinal-Ang IV (10^-5^ M), FoxO1-overexpression plasmid (FoxO1-OE; Gene ID: 84482; Genechem, Shanghai, China), AS (1 μM), Ang IV plus FoxO1-OE, and Ang IV plus divalinal-Ang IV plus FoxO1-OE were respectively added to cardiomyocytes and cardiac fibroblasts under a glucose concentration of 60 mM. FoxO1-OE plasmid and empty vector were transfected into cells by Lipo3000 (L3000015, Invitrogen, Carlsbad, CA) under standard procedures as indicated in the manufacturer's protocol, and 24 h after transfection, other agents were added into cell medium 24 h before harvest.

### Statistical analysis

Continuous data were expressed as mean ± standard error (SEM). After testing for normality and equality of variance, continuous data were compared by unpaired Student's t-test or one-way analysis of variance (ANOVA). Post hoc Tukey tests were performed where significant interactions were observed in ANOVAs. The Benjamini-Hochberg method was used for multiple test correction. Survival analysis was performed using Kaplan-Meier curves and log-rank test. Statistical analysis was performed using SPSS v21.0 (SPSS Inc., Chicago, IL) and a two-tailed *p* < 0.05 was considered statistically significant.

## Results

### Ang IV treatment improved left ventricular dysfunction and remodeling in diabetic mice

Serum lipid levels, blood pressure and mortality rate exhibited no significant difference among 5 groups of mice in the first part of the *in vivo* experiment (NC, DM, DM+low-dose Ang IV, DM+medium-dose Ang IV, and DM+high-dose Ang IV) (Figure S1A and Table S1). FBG was significantly increased in the 4 groups of mice with diabetes relative to the NC group but did not differ among DM, DM+low-dose Ang IV, DM+medium-dose Ang IV, and DM+high-dose Ang IV groups (Table S1).

Echocardiographic measurements of left ventricular ejection fraction (LVEF), fractional shortening (FS), the ratio of the early to late diastolic mitral inflow velocities (E/A) and the ratio of the early to late diastolic mitral annular velocities (E'/A') were significantly decreased whereas left ventricular end-diastolic diameter (LVEDD) was increased in diabetic mice compared to those in the NC group at the end of 24 weeks (Figure 1C and Table 1). Ang IV treatment for 16 weeks dose-dependently increased values of LVEF, FS, E/A and E'/A' and decreased that of LVEDD relative to the DM group (Figure 1C and Table 1).

**Figure 2:**
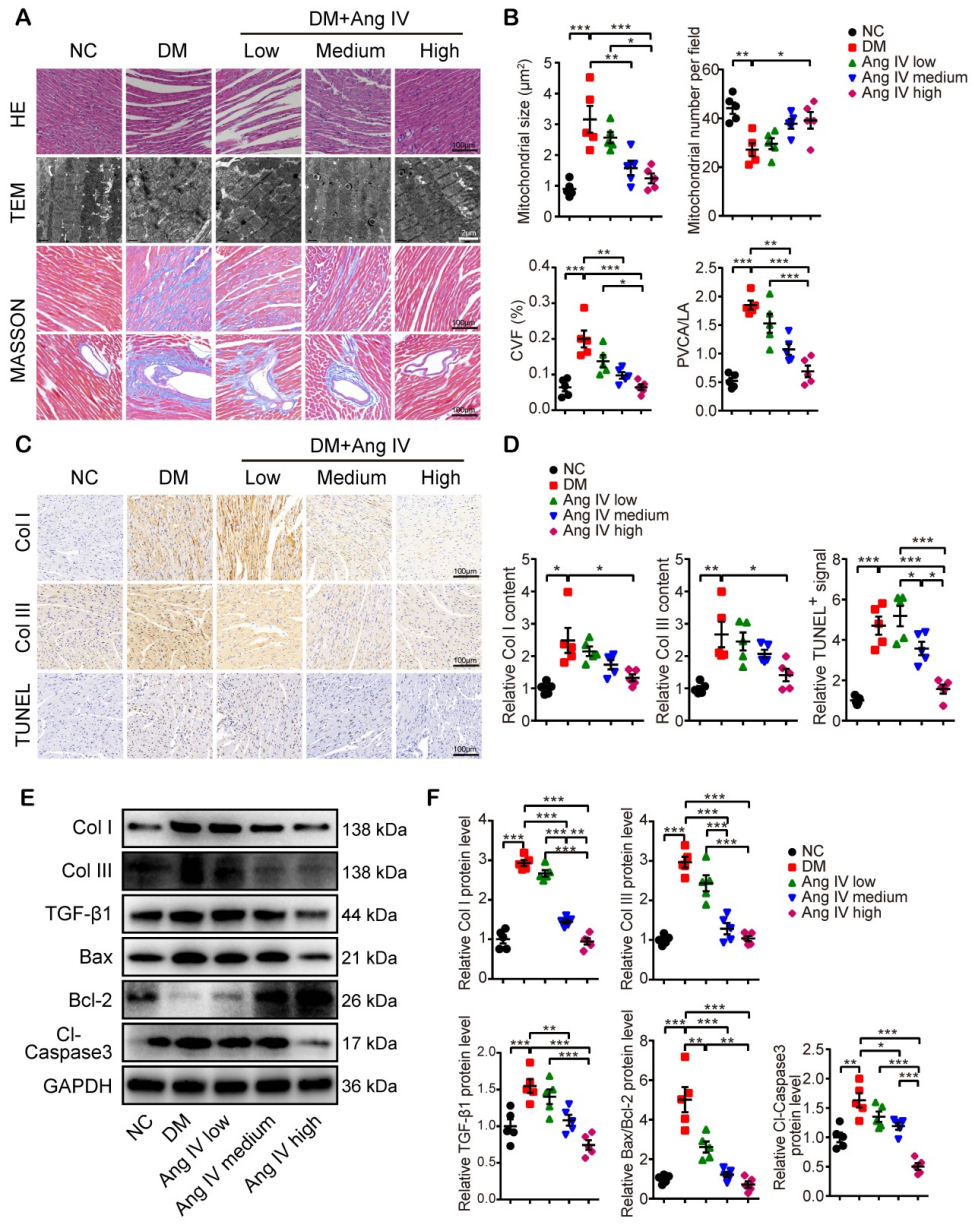
** Effects of Ang IV on myocardial morphology, fibrosis and apoptosis in 5 groups of mice. (A**) Representative images of H&E staining, TEM, and Masson's trichrome staining of the myocardium in 5 groups of mice. **(B)** Quantification of mitochondrial size, mitochondrial number per field, CVF and PVCA/LA in 5 groups of mice.** (C)** Representative immunohistochemical staining of Col I, Col III, and TUNEL in the myocardium of 5 groups of mice. **(D)** Quantification of immunohistochemical staining of Col I, Col III, and TUNEL in the myocardium of 5 groups of mice.** (E)** Representative Western blot images of Col I, Col III, TGF-β1, Bax, Bcl-2 and Cl-caspase3 expressions in the myocardium of the 5 groups of mice. **(F)** Quantification of the protein expressions of Col I, Col III, TGF-β1, Bax/Bcl-2 and Cl-caspase3 in the myocardium of the 5 groups of mice. n=5 per group. Ang IV: angiotensin IV; Cl-caspase3: cleaved caspase 3; Col I: collagen I; Col III: collagen III; DM: diabetes mellitus; NC: normal control. *p < 0.05, **p < 0.01, ***p < 0.001.

Morphologically, mice of the DM group exhibited significant myocardial disarray under H&E staining, as well as disordered myofibrils and swollen mitochondria under transmission electron microscopy (TEM), accompanied by increased mitochondrial size and reduced mitochondrial number per field, as compared with mice of the NC group (Figure 2A-2B). In contrast, these abnormalities in myocardial morphology and ultrastructure were largely reversed by Ang IV treatment, especially in mice of the DM+high-dose Ang IV group (Figure 2A). Quantitative analyses of collagen volume fraction (CVF) and perivascular collagen area to luminal area (PVCA/LA) by Masson's trichrome staining revealed significant collagen deposition in the myocardium of diabetic mice versus the NC group, which was substantially alleviated by Ang IV treatment, particularly in the DM+high-dose Ang IV group (Figure 2A-2B). Similarly, immunohistochemical staining showed dramatic upregulation of collagen I (Col I) and collagen III (Col III) in the myocardium of diabetic mice relative to the NC group, which was downregulated dose-dependently by Ang IV treatment (Figure 2C-2D). We also detected the apoptosis in the myocardium by TUNEL staining, which revealed an upsurge of TUNEL positive signals in the myocardium of diabetic mice compared with the NC group, and this abnormality was effectively normalized by high-dose Ang IV treatment (Figure 2C-2D).

Previous studies showed that Col I, Col III and transforming growth factor-β 1 (TGF-β1) were highly expressed in fibrotic myocardium, which have been recognized as hallmarks of collagen deposition and myocardial remodeling. In our experiments, Western blot analyses of myocardial tissues indicated that the protein expressions of Col I, Col III and TGF-β1 were significantly higher in the DM than the NC mice, which were lowered dose-dependently by Ang IV treatment (Figure 2E-2F). The Bax/Bcl-2 protein ratio and cleaved caspase 3 (Cl-caspase3) protein level were elevated in the myocardium of DM mice versus those of NC mice, which were normalized by medium- or high-dose Ang IV treatment (Figure 2E-2F). These results suggested that Ang IV treatment dose-dependently attenuated cardiac dysfunction, fibrosis, and apoptosis in diabetic mice.

### Ang IV treatment suppressed overactive autophagy and FoxO pathway in diabetic mice

**Figure 3:**
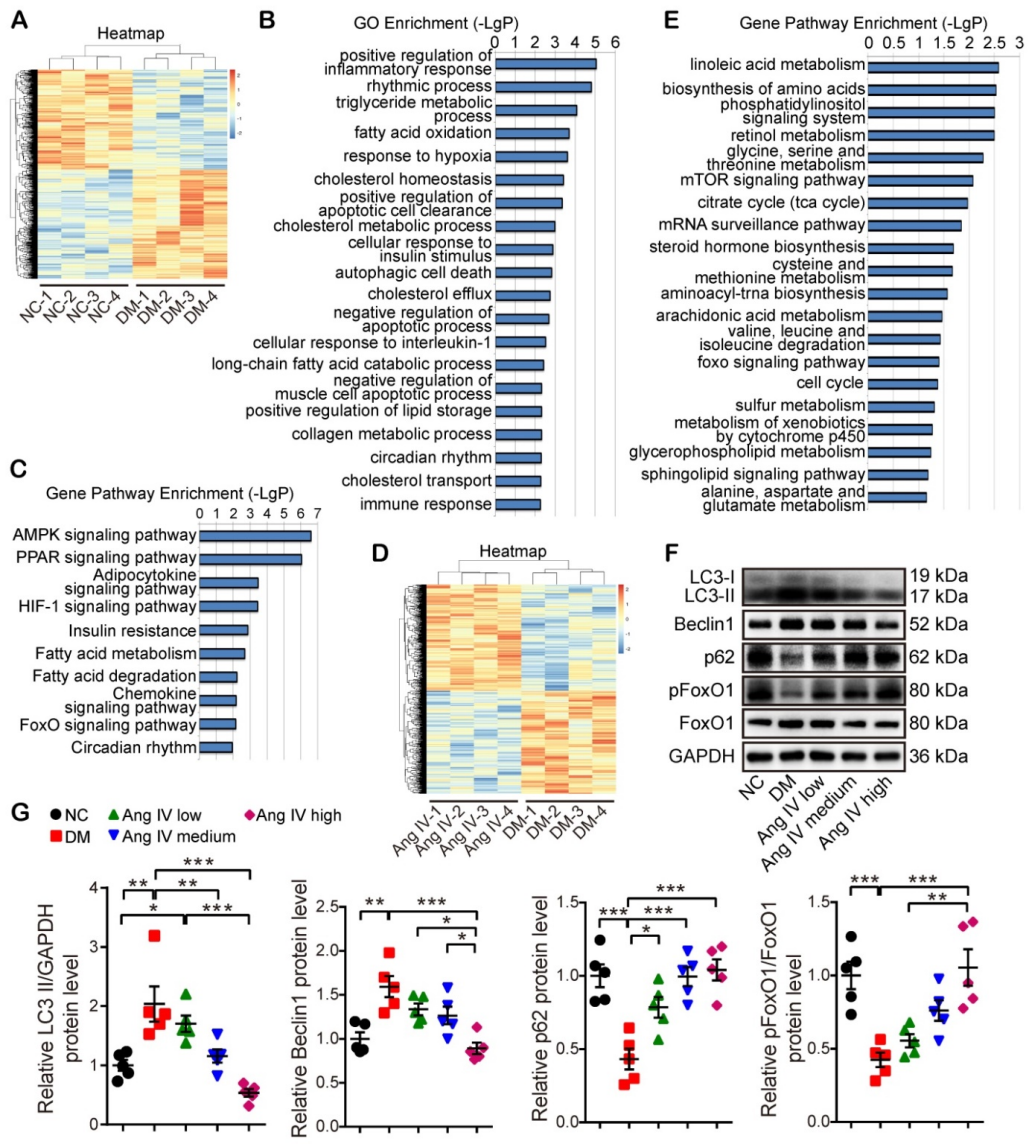
** Transcriptome profiles of the myocardium in 3 groups of mice and effects of Ang IV on the expressions of autophagy-associated proteins in the myocardium of 5 groups of mice. (A)** Hierarchical clustering heatmap to identify differentially expressed genes in NC and DM groups of mice. **(B)** The top 20 biological processes from the GO enrichment analysis of differentially expressed genes in NC and DM groups of mice. **(C)** The top 10 gene pathways from KEGG pathway enrichment analysis of differentially expressed genes in NC and DM groups of mice. **(D)** Hierarchical clustering heatmap to identify differentially expressed genes in DM and DM+high-dose Ang IV groups of mice. **(E)** The top 20 gene pathways from KEGG pathway enrichment analysis of differentially expressed genes in DM and DM+high-dose Ang IV groups of mice. n=4 per group. **(F)** Representative Western blot images of LC3, Beclin1, p62, pFoxO1 and FoxO1 protein expression in the myocardium of the 5 groups of mice. **(G)** Quantification of LC3-II, Beclin1, p62 and pFoxO1/FoxO1 protein expression in the myocardium of the 5 groups of mice. n=5 per group. Ang IV: angiotensin IV; DM: diabetes mellitus; NC: normal control. *p < 0.05, **p < 0.01, ***p < 0.001.

To reveal the mechanism underlying the therapeutic effect of Ang IV in diabetic mice, 4 mice from each of the NC, DM and DM+high-dose Ang IV groups were randomly selected in whom RNA sequencing assay in the myocardium was performed. The gene expression profiles within the 4 mice in each of the three groups were highly reproducible (Figure 3A, 3D). Gene Ontology (GO) enrichment revealed that among the top 20 biological processes detected in NC and DM mice, inflammatory response, lipid and glucose metabolism, hypoxia response, collagen metabolism, autophagy and apoptosis were mostly involved (Figure 3B). Gene pathway enrichment demonstrated that among the top 10 signaling pathways screened from the 2 groups of mice, AMPK, PPAR, HIF-1 and FoxO pathways were most evident (Figure 3C). Moreover, gene pathway enrichment showed that the mTOR and FoxO pathways were involved in the top 20 signaling pathways screened from the DM and DM+high-dose Ang IV mice (Figure 3E). As mTOR acts as a brake on autophagy and the FoxO pathway is a key regulator of autophagy 21, we focused on autophagy and the FoxO pathway in the present study.

To examine autophagy flux changes induced by DM *in vivo*, mice in the NC and DM groups were administered with chloroquine before euthanasia. As chloroquine inhibits lysosomal activity, the number of monodansyl cadaverine (MDC) labelled particles, a marker of autophagosomes 22, was remarkably increased in the myocardium of DM mice relative to that of the NC group (Figure S2A-2B). Moreover, the protein expression level of LC3-II was significantly higher whereas that of p62 was lower in DM mice than in the NC group after chloroquine treatment (Figure S2C-2D), suggesting that DM may induce overactive autophagy. We then examined the protein expression levels of several autophagy-related proteins including LC3, Beclin1, and p62 in the myocardium of the mice treated with Ang IV at different doses. Western blot revealed that the protein expressions of LC3-II and Beclin1 were significantly increased whereas that of p62 decreased in the DM group in comparison with the NC group, and these abnormalities were dose-dependently reversed by Ang IV treatment, especially in the DM+high-dose Ang IV group, indicating that Ang IV dose-dependently attenuated overactive autophagy in the myocardium of diabetic mice (Figure 3F-3G). Further experiments showed that the protein expression levels of LC3-II and p62 in the myocardium exhibited no significant difference between normal mice treated with high-dose Ang IV and those treated with vehicle (Figure S2E-2F). These results indicated that Ang IV did not inhibit autophagy in healthy hearts, but reversed excessive autophagy in diabetic hearts.

We then assessed the protein expression and phosphorylation levels of FoxO1, a key effector in the FoxO pathway. FoxO1 is translocated from the nucleus to the cytoplasm and degraded once phosphorylated, thus losing its ability to regulate the target genes. Western blot revealed that pFoxO1/FoxO1 ratio was significantly decreased in DM mice relative to the NC mice, and high-dose Ang IV treatment normalized pFoxO1/FoxO1 ratio in the DM mice (Figure 3F-3G), suggesting that Ang IV inhibited DM-activated FoxO1 signaling pathway.

### AT_4_R played a central role in the therapeutic effects of Ang IV

**Figure 4:**
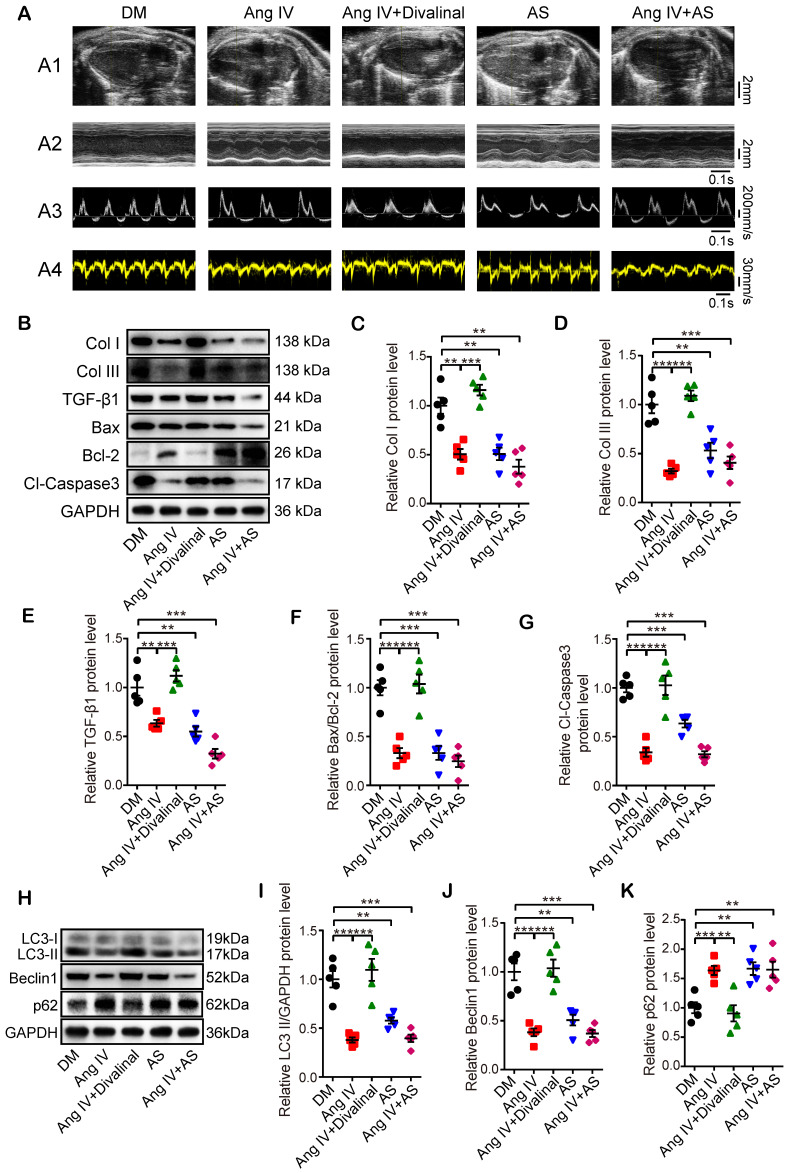
** Effects of AT_4_R and FoxO1 on left ventricular function and expressions of fibrosis-, apoptosis- and autophagy-associated proteins in the myocardium of 5 groups of mice. (A)** Representative echocardiographic images in 5 groups of mice. **(A1)** Two-dimensional echocardiograms showing left ventricular long-axis views and scale bar in mm on the right; **(A2)** M-mode echocardiograms showing left ventricular dimensions, scale bar in mm on the right, and time stamp in seconds at the bottom; **(A3)** Pulse-wave Doppler echocardiograms depicting mitral inflow velocities, scale bar in mm/s on the right, and time stamp in seconds at the bottom; **(A4)** Tissue Doppler echocardiograms displaying mitral annular velocities, scale bar in mm/s on the right, and time stamp in seconds at the bottom. n≥8 per group. **(B)** Representative Western blot images of Col I, Col III, TGF-β1, Bax, Bcl-2 and Cl-caspase3 protein expressions in the myocardium of 5 groups of mice. **(C-G)** Quantifications of Col I, Col III, TGF-β1, Bax/Bcl-2 and Cl-caspase3 protein expressions in the myocardium of 5 groups of mice. **(H)** Representative Western blot images of LC3, Beclin1 and p62 expressions in the myocardium of 5 groups of mice. **(I-K)** Quantification of LC3-II, Beclin1 and p62 expressions in the myocardium of 5 groups of mice. After DM was successfully induced, mice were divided into the following 5 groups: DM group that received an infusion of vehicle alone, Ang IV group that received an infusion of high-dose Ang IV, Ang IV+divalinal group that received an infusion of high-dose Ang IV plus AT_4_R antagonist divalinal, AS group that received an infusion of FoxO1 inhibitor AS1842856, and Ang IV+AS group that received an infusion of Ang IV plus AS1842856. n=5 per group. Ang IV: angiotensin IV; AS: AS1842856; Cl-caspase3: cleaved caspase 3; Col I: collagen I; Col III: collagen III. *p < 0.05, **p < 0.01, ***p < 0.001.

To clarify the role of AT_4_R in the therapeutic effects of Ang IV in diabetic mice, we used a specific AT_4_R antagonist divalinal-Ang IV and a specific FoxO1 inhibitor AS in the second part of the *in vivo* experiment. Our results showed that there was no significant difference in serum lipid profile, FBG and blood pressure and mortality rate among DM, Ang IV, Ang IV+divalinal, AS, and Ang IV+AS groups (Figure S1B and Table S2). Echocardiographic measurements of LVEF, FS, E/A and E'/A' were significantly increased whereas that of LVEDD decreased in the Ang IV and the AS groups relative to the DM group at the end of 24 weeks (Figure 4A and Table 2). In contrast, these beneficial effects of Ang IV were abolished in the Ang IV+divalinal group. Treatment with both Ang IV and AS did not further enhance the cardioprotective effects of either single therapy (Figure 4A and Table 2). These results indicated that Ang IV and FoxO1 inhibitor exerted similar salutary effects on left ventricular dysfunction and remodeling in diabetic mice, and the effects of Ang IV were mediated *via* AT_4_R.

Masson's trichrome staining, Western blot and histochemical staining showed that measurements of CVF and PVCA/LA, and protein expressions of Col I, Col III and TGF-β1 were substantially decreased in the Ang IV and the AS groups compared with the DM group. On the other hand, these cardioprotective effects of Ang IV were largely offset by the addition of divalinal to Ang IV treatment. The combined administration of Ang IV and AS yielded no further benefits (Figure 4B-4E and Figure S3A-3F). These results suggested that treatment with Ang IV or FoxO1 inhibitor improved myocardial fibrosis in diabetic mice, and the anti-fibrotic effects of Ang IV were mediated *via* AT_4_R. Similarly, Bax/Bcl-2 protein ratio, Cl-caspase3 expression level (Figure 4B, 4F-4G), and TUNEL positive signals in the myocardium (Figure S3D, S3G) were dramatically reduced in the Ang IV and the AS groups versus DM group, and the protective effects of Ang IV were virtually eliminated by the addition of divalinal to Ang IV treatment. Besides, the protein expressions of LC3-II and Beclin1 were lower while that of p62 was higher in the AS group than the DM group, indicating that FoxO1 is one key factor in regulating myocardial fibrosis, apoptosis and autophagy in diabetic mice (Figure 4H-4K). All these results indicated that the cardioprotective effects of Ang IV depended on both AT_4_R and the FoxO1 pathway.

### Ang IV inhibited high glucose-enhanced expressions of fibrosis- and apoptosis-associated markers and autophagy flux *in vitro*

In the first part of the* in vitro* experiment, H9C2 cardiomyocytes were treated with different concentrations of glucose*.* The results showed that the LC3-II/GAPDH and Bax/Bcl-2 ratios were higher in cardiomyocytes treated with 60 mM glucose than those with 5.5, 15, 25 or 33.3 mM glucose, while the protein expression of p62 was lowest in cardiomyocytes treated with 60 mM glucose (Figure S4).

**Figure 5:**
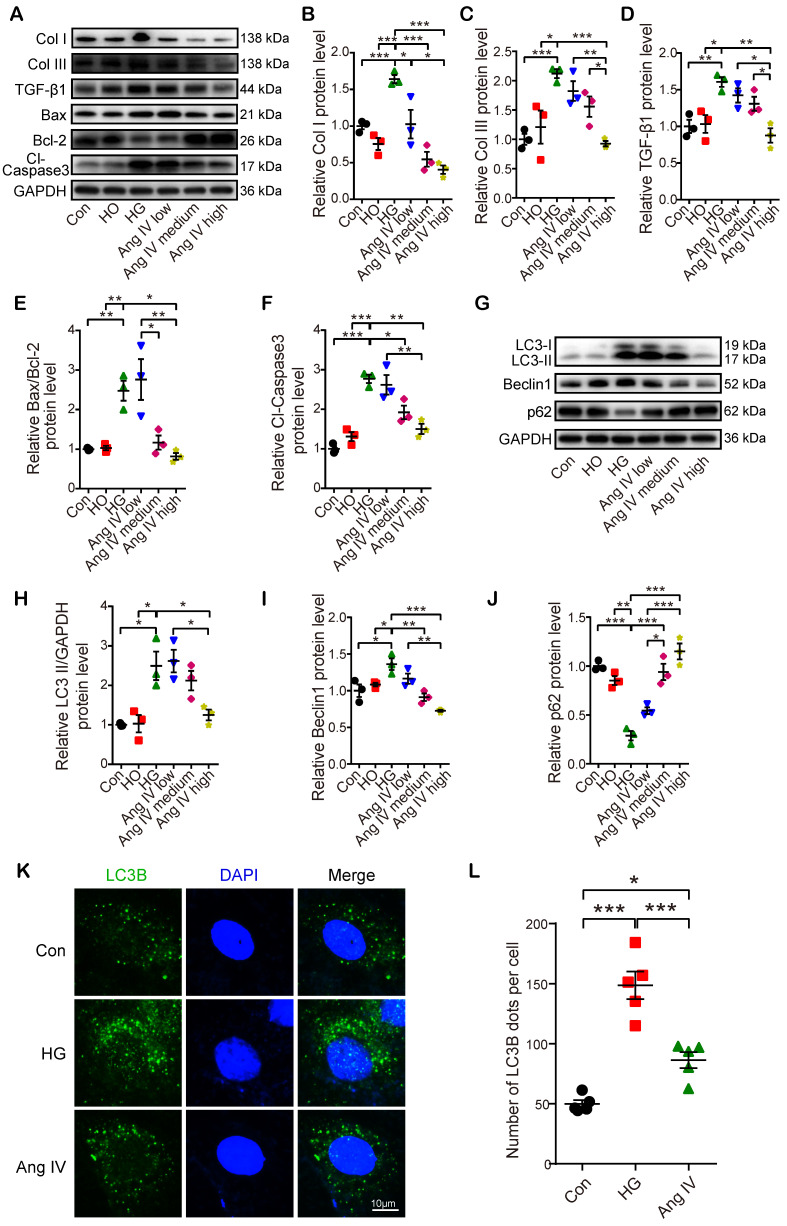
** Effects of Ang IV on the expressions of fibrosis-, apoptosis- and autophagy-associated markers and autophagy flux in cardiomyocytes. (A)** Representative Western blot images of Col I, Col III, TGF-β1, Bax, Bcl-2 and Cl-caspase3 in 6 groups of cells. **(B-F)** Quantification of Col I, Col III, TGF-β1, Bax/Bcl-2 and Cl-caspase3 expressions in 6 groups of cells. **(G)** Representative Western blot images of LC3, Beclin1 and p62 in 6 groups of cells. **(H-J)** Quantification of LC3-II, Beclin1 and p62 expressions in 6 groups of cells. n=3 per group. **(K)** Representative images of immunofluorescent staining of LC3B in 3 groups of cells. **(L)** Quantification of immunofluorescent staining of LC3B in 3 groups of cells. n=5 per group. Ang IV: angiotensin IV; Cl-caspase3: cleaved caspase 3; Con: normal glucose control; Col I: collagen I; Col III: collagen III; HG: high glucose; HO: high osmotic control. *p < 0.05, **p < 0.01, ***p < 0.001.

In the second part of the* in vitro* experiment, cardiomyocytes and cardiac fibroblasts were treated with vehicle or Ang IV in 60 mM glucose solution. The expression levels of fibrosis- (Col I, Col III, and TGF-β1), apoptosis- (Bax, Bcl-2, and Cl-caspase3) and autophagy-related proteins (LC3-II, Beclin1, and p62) were quantitated by Western blot. The results showed that protein expressions of Col I, Col III and TGF-β1 in cardiomyocytes and cardiac fibroblasts were significantly increased in the HG group relative to the Con and the HO groups, while such differences were dose-dependently lessened by Ang IV administration, and was virtually eliminated in the high-dose Ang IV group (Figure 5A-5D and Figure S5A-5D), a finding consistent with the *in vivo* experiment. Similarly, in cardiomyocytes, Bax/Bcl-2 protein ratio and Cl-caspase3 protein expression were substantially increased in the HG group versus the Con and the HO groups, which were dose-dependently downregulated by Ang IV treatment (Figure 5A, 5E-5F). These results suggested that Ang IV effectively counteracted HG-enhanced expressions of collagen and TGF-β1 in cardiomyocytes and cardiac fibroblasts, as well as those of Bax/Bcl-2 and Cl-caspase3 in cardiomyocytes, which lent support to the anti-fibrosis and anti-apoptosis effects of Ang IV *in vivo*. Besides, the expressions of LC3-II and Beclin1 in cardiomyocytes were significantly upregulated in the HG group in comparison with the Con and the HO groups, while such upregulation was dose-dependently attenuated by Ang IV treatment. On the other hand, the expression of p62 showed opposite changes among the 5 groups of cardiomyocytes (Figure 5G-5J). LC3B was also stained by immunofluorescence assay after chloroquine administration which showed that high-dose Ang IV treatment suppressed autophagy flux enhanced by high glucose in cardiomyocytes (Figure 5K-5L). Taken together, these results demonstrated that Ang IV treatment dose-dependently reversed the high glucose-enhanced expressions of fibrosis-, apoptosis- and autophagy-related proteins, thus protecting cardiomyocytes from death *via* inhibiting excessive autophagy.

### FoxO1 acted as a mediator between the Ang IV-AT_4_R axis and cardioprotection *in vitro*

**Figure 6:**
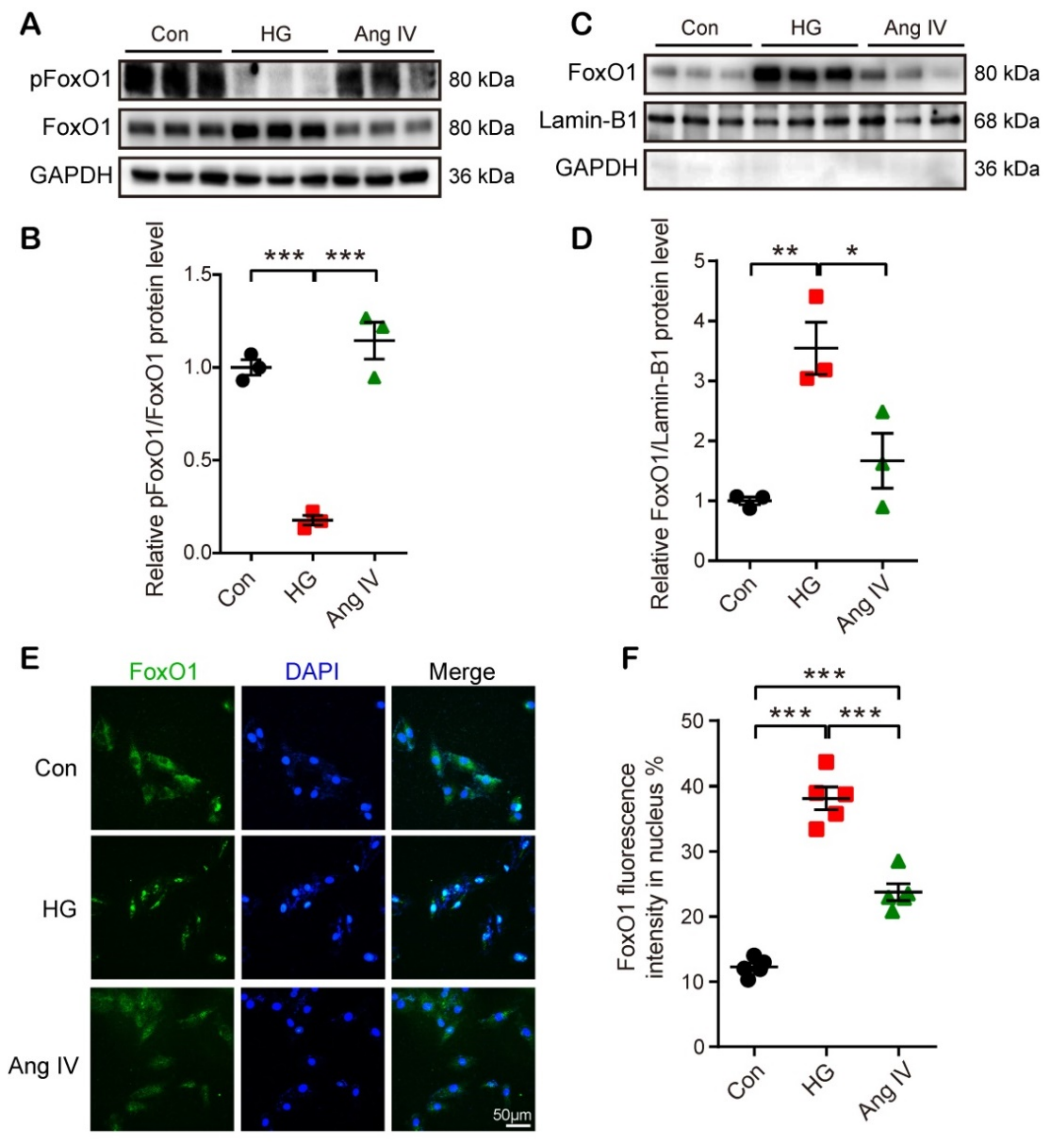
** Effects of Ang IV on FoxO1 phosphorylation and nuclear translocation in cardiomyocytes. (A)** Representative Western blot images of pFoxO1 and FoxO1 expression in 3 groups of cells. **(B)** Quantification of pFoxO1/FoxO1 ratio in 3 groups of cells. n=3 per group.** (C)** Representative Western blot images of nuclear FoxO1 and nuclear internal control expressions in 3 groups of cells. **(D)** Quantification of nuclear FoxO1 in 3 groups of cells. n=3 per group. **(E)** Representative images of immunofluorescent staining of FoxO1 in 3 groups of cells. **(F)** Quantification of immunofluorescent staining of FoxO1 in 3 groups of cells. n=5 per group. Ang IV: angiotensin IV; Con: normal glucose control; HG: high glucose. *p < 0.05, **p < 0.01, ***p < 0.001.

FoxO1 is primarily regulated through phosphorrylation and the nuclear import of dephosphorylated FoxO1 is a key step to regulate transcriptions of downstream target genes, especially autophagy-related genes *Lc3b* and* Becn1* 23. Hence, we detected FoxO1 phosphorylation and nuclear translocation by Western blot and immunofluorescence staining under Con, HG, and high-dose Ang IV conditions. The results showed that the FoxO1 phosphorylation level was dramatically reduced and the nuclear expression of FoxO1 in cardiomyocytes was significantly increased in the HG group relative to the Con group, and this effect was offset by high-dose Ang IV administration (Figure 6A-6D). Likewise, about 12% of FoxO1 maintained in the nucleus under normal condition, and this percentage increased to 39% under HG condition but declined to 24% after high-dose Ang IV treatment (Figure 6E-6F). These results strongly suggested that Ang IV treatment promoted FoxO1 phosphorylation and suppressed FoxO1 nuclear translocation induced by HG stimulation. Similarly, Ang IV treatment dose-dependently inhibited the HG-induced FoxO1 expression in cardiac fibroblasts (Figure S5A, S5E).

**Figure 7:**
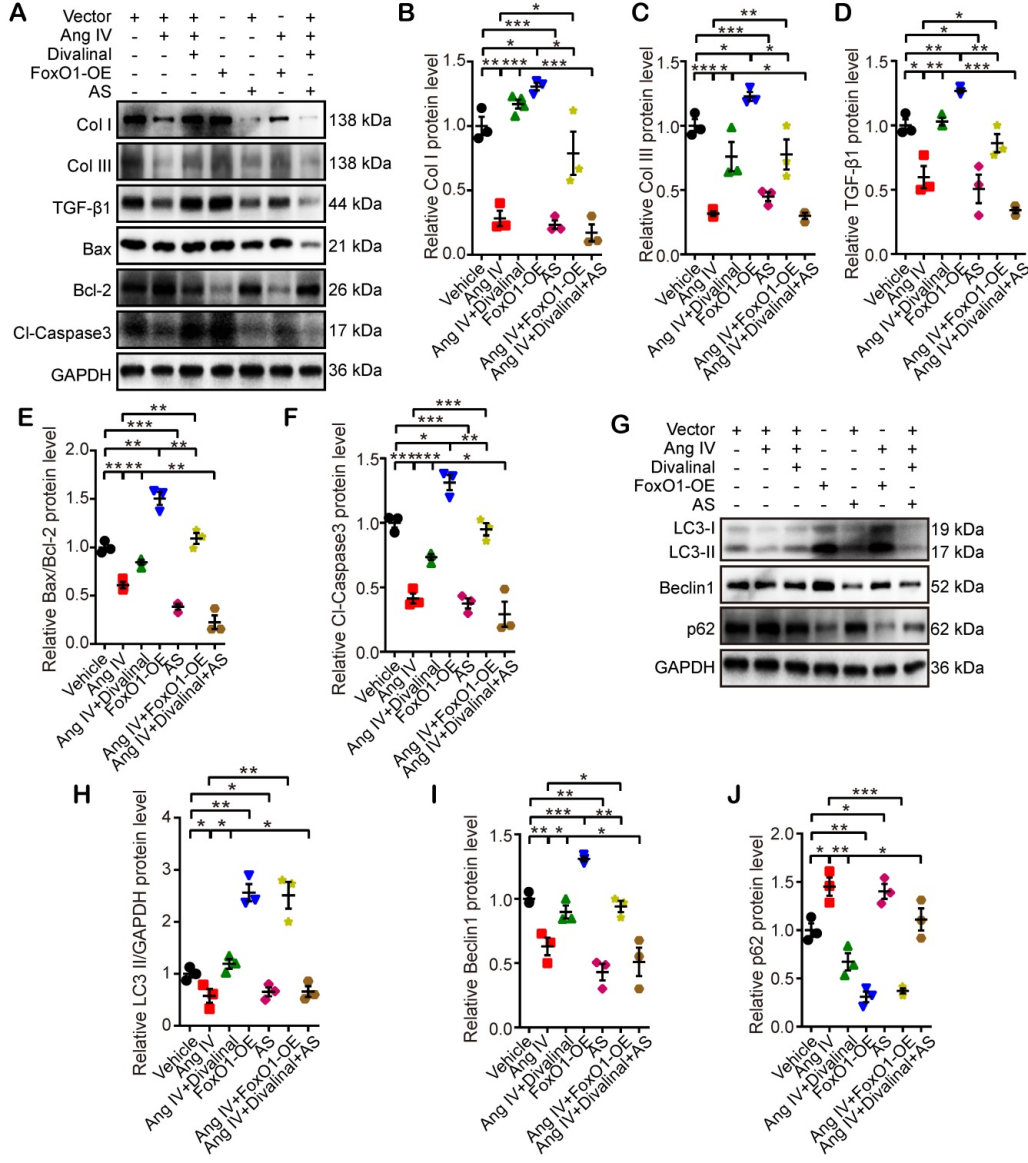
** Effects of FoxO1 and AT_4_R on the expressions of fibrosis-, apoptosis- and autophagy-associated proteins in cardiomyocytes. (A)** Representative Western blot images of Col I, Col III, TGF-β1, Bax, Bcl-2 and Cl-caspase3 in 7 groups of cells. **(B-F)** Quantification of Col I, Col III, TGF-β1, Bax/Bcl-2 and Cl-caspase3 expressions in 7 groups of cells. **(G)** Representative Western blot images of LC3, Beclin1 and p62 in 7 groups of cells. **(H-J)** Quantification of LC3-II, Beclin1 and p62 expressions in 7 groups of cells. n=3 per group. Ang IV: angiotensin IV; AS: AS1842856; Cl-caspase3: cleaved caspase 3; Col I: collagen I; Col III: collagen III; FoxO1-OE: FoxO1 overexpression. *p < 0.05, **p < 0.01, ***p < 0.001.

In the third part of the *in vitro* experiment, cardiomyocytes and cardiac fibroblasts were stimulated by Ang IV, divalinal-Ang IV, AS, FoxO1-OE, or their combinations under HG condition. The expression levels of fibrosis- (Col I, Col III, and TGF-β1), apoptosis- (Bax, Bcl-2, and Cl-caspase3) and autophagy-related proteins (LC3-II/GAPDH, Beclin1, and p62) were quantitated by Western blot. The expression levels of Col I, Col III and TGF-β1 in cardiomyocytes and cardiac fibroblasts were significantly reduced in the Ang IV group relative to the vehicle group, and such effects were completely offset by co-administration of divalinal and Ang IV (Figure 7A-7D and Figure S5F-S5I). Similarly, Col I, Col III and TGF-β1 expressions were upregulated in the FoxO1-OE group versus the vehicle group while such upregulation was entirely counteracted by the administration of AS (Figure 7A-7D and Figure S5F-S5I). It is interesting to note that the beneficial effects of Ang IV on the HG-enhanced expressions of Col I, Col III and TGF-β1 were largely reversed in the Ang IV+FoxO1-OE group relative to the Ang IV group, and more importantly, the expressions of Col I, Col III and TGF-β1 were substantially lower in the Ang IV+divalinal+AS group than the Ang IV+divalinal group. These results strongly suggested that FoxO1 might be a downstream effector of the Ang IV-AT_4_R axis after HG stimulation. The alterations of the expression levels of apoptosis-related proteins (Bax/Bcl-2 and Cl-caspase3) showed the same trend as fibrosis-related proteins (Col I, Col III, and TGF-β1) among the 7 groups of cardiomyocytes (Figure 7A, 7E-7F), further demonstrating that FoxO1 mediated the anti-apoptosis effect of the Ang IV-AT_4_R axis under HG condition. Likewise, the alterations of the expression levels of autophagy-related proteins (LC3-II and Beclin1) showed the same trend as fibrosis- and apoptosis-related proteins among the 7 groups of cardiomyocytes (Figure 7G-7I), while the expression of p62 exhibited opposite changes as compared with LC3-II and Beclin1 (Figure 7G, 7J). The proposed mechanism underlying the therapeutic effects of Ang IV in our mouse model of diabetic cardiomyopathy was summarized in the graphic abstract.

## Discussion

There were several important findings in the present study. First, Ang IV treatment dose-dependently attenuated left ventricular dysfunction, remodeling, fibrosis, and myocyte apoptosis in a mouse model of diabetic cardiomyopathy. Second, AS1842856, a specific FoxO1 inhibitor, had a similar therapeutic effect on diabetic cardiomyopathy. Third, the cardioprotective effects of Ang IV were abolished by a combined administration of divalinal, a specific AT_4_R antagonist, or by combinational use of FoxO1 overexpression. Fourth, autophagy over-activation was involved in the pathogenesis of diabetic cardiomyopathy, which was effectively inhibited by Ang IV treatment. Finally, FoxO1 inhibition and large-dose Ang IV had no synergistic effect on diabetic cardiomyopathy, but FoxO1 inhibition completely reversed the detrimental effects of divalinal administration. To the best of our knowledge, our study is the first in the literature to report the therapeutic effects of Ang IV on diabetic cardiomyopathy and the potential mechanisms involved.

RAAS conventionally refers to the ACE-Ang II-AT_1_R axis, which is activated in a variety of cardiovascular diseases including diabetic cardiomyopathy associated with autophagy disorders 17, 18, 24. Although the detrimental effects of the ACE-Ang II-AT_1_R axis can be effectively opposed by ACE inhibitors or AT_1_R blockers, the therapeutic efficacy of these medications is far from being satisfactory. Our previous studies demonstrated that ACE2 overexpression alleviated left ventricular remodeling and dysfunction induced by type 1 diabetes or acute myocardial infarction in rats, which was accompanied by downregulated Ang II and upregulated Ang-(1-7) levels 25, 26. Moreover, we revealed that Ang-(1-7) dose-dependently diminished left ventricular fibrosis, hypertrophy, apoptosis, and dysfunction, as well as right ventricular fibrosis and dysfunction, *via* a complex interaction of MasR, AT_2_R and AT_1_R, in diabetic rats 8, 9. Besides the ACE2-Ang-(1-7)-MasR/AT_2_R axis, we further explored the role of Ang IV in Ang II-induced abdominal aortic aneurysm (AAA) in apolipoprotein E-knockout (ApoE^-/-^) mice and found dose-dependent bidirectional effects of Ang IV: a medium-dose Ang IV (1.44 mg/kg daily) significantly reduced the incidence and severity of AAA, whereas a high-dose Ang IV (2.88 mg/kg daily) had no protective effect. The mechanism may involve variable angiotensin receptor stimulation. The beneficial effect of medium-dose Ang IV may be contributed to the stimulation of AT_4_R and AT_2_R and suppression of AT_1_R, whereas Ang IV may act as an agonist of AT_1_R at a high dose 27. In the current study, we found that Ang IV dose-dependently attenuated diabetic cardiomyopathy, with the best efficacy being noted in the large-dose group (2.88 mg/kg daily). On the other hand, antagonism of AT_4_R by divalinal completely abolished the protective effect of high-dose Ang IV, indicating the effect of Ang IV on diabetic cardiomyopathy was mediated essentially *via* AT_4_R. These results were consistent with a previous report that Ang IV treatment attenuated ischemia/reperfusion-induced cardiac injury, and this salutary effect was blunted by AT_4_R antagonist but not by AT_1_R, AT_2_R or MasR antagonists 14. The reason for this discrepancy in the dose-response relationship between AAA and diabetic cardiomyopathy may be multiple. First, AT_1_R is highly activated in the AAA mice after a long-term infusion of a very high dose of Ang II and may exhibit a higher affinity with Ang IV than in the current model. Second, the serum lipid level in the ApoE^-/-^ mice with AAA is much higher than that in the diabetic mice of this study, which showed no difference from normal mice (Table S1). Finally, the expression and activity of AT_1_R, AT_2_R and AT_4_R may be different between the abdominal aorta and myocardium. Our results showed that Ang IV treatment did not affect serum lipid, glucose and blood pressure levels, indicating that the beneficial effect of Ang IV on diabetic cardiomyopathy cannot be attributed to metabolic and hemodynamic changes, which was consistent with our previous results of Ang-(1-7) treatment in a mouse model of diabetic cardiomyopathy 8, 9.

Autophagy is a critical route of cell death and helps maintain cellular homeostasis and housekeeping functions in the heart at the basal level 15, 16. However, overactive autophagy in hypertrophied or ischemic heart aggravated myocardial apoptosis and fibrosis and induced the transition from myocardial hypertrophy or ischemia/reperfusion injury to heart failure 28, 29. However, the precise role of myocyte autophagy and its molecular mechanisms in diabetic cardiomyopathy are still controversial. Some studies showed that diabetic cardiomyopathy was associated with down-regulation of myocyte autophagy, whereas induction of autophagy mitigated cardiac dysfunction and myocyte apoptosis in diabetic mice 30-32. In contrast, other studies presented the opposite results 33, 34. Besides, autophagy may play paradoxical roles during different pathological processes. For instance, autophagy is protective during ischemia but detrimental during reperfusion 28. The controversial results might attribute to the different animal models, blood glucose levels, and durations of diabetes. In the present study, the diabetic murine model was induced by multiple intraperitoneal injections of STZ at a dosage of 55 mg/kg daily for 5 consecutive days, and a long course of diabetes (FBG ≥16.7 mM over 6 months) induced marked left ventricular dilation and systolic and diastolic dysfunction, a condition mimicking human diabetic cardiomyopathy. Consistent with our animal model, cardiomyocytes were incubated with glucose in 5% fetal bovine serum to minimize the effect of insulin in the bovine serum. Our studies demonstrated that overactive autophagy occurred in the myocardium of diabetic mice and high glucose enhanced autophagy flux in cardiomyocytes. Thus, overactive autophagy plays a pivotal role in the pathogenesis of diabetic cardiomyopathy that may serve as a potential interventional target for diabetes-induced myocardial remodeling and dysfunction.

One major finding of the present study was that Ang IV treatment dose-dependently attenuated diabetic cardiomyopathy *via* down-regulating FoxO1-mediated autophagy, which was supported by our *in vitro* result that Ang IV promoted FoxO1 phosphorylation and suppressed FoxO1 nuclear translocation induced by HG in cardiomyocytes. Moreover, myocardial autophagy was attenuated by FoxO1 inhibition or Ang IV administration, and enhanced by FoxO1 overexpression regardless of combined Ang IV treatment. These results suggested that FoxO1 may be both a downstream effector of the Ang IV-AT_4_R axis and an upstream trigger of the autophagy process, thus serving as a key node in the mechanistic network of the diabetic cardiomyopathy. FoxO1 was reported to directly regulate autophagy in a transcription-independent manner, and acetylated FoxO1 could induce autophagy by interaction with autophagy-related protein 7 in cancer cells 35. In adipocytes, FoxO1 regulated lipid droplet growth and size by enhancing autophagy, and FoxO1 inhibition reduced the expression of Beclin1 which increased the stability of p62, an inhibitor of autophagy 36. Our observation of swollen mitochondria in DM mice is probably a secondary effect caused by metabolism imbalances. In the rat heart with STZ-induced diabetes, FoxO1 overactivation was found to shift substrate selection from glucose to fatty acid, induce disarranged oxidative metabolism, mitochondrial dysfunction and cardiomyocyte apoptosis, and ultimately cause cardiac dysfunction 37. FoxO1 was also reported to function together with putative kinase 1 (PINK1) to mediate mitochondrial oxidative stress 38. The accumulation of PINK1 on the mitochondrial outer membrane triggers the recruitment of Parkin to the mitochondria, leading to autophagic degradation of the dysfunctional mitochondrion 38. A recent study revealed that phosphorylated (inactive) FoxO1 level was reduced and the nuclear localization of FoxO1 was increased in the mouse heart with laminopathies. Suppression of activated FoxO transcription factors in cardiomyocytes prolonged mouse survival 39. The relationship between Ang IV and mitochondria has not been reported but our results suggest that Ang IV may rescue mitochondrial damage *via* promoting FoxO1 phosphorylation in diabetic hearts. Thus, inhibition of FoxO1 may provide an alternative approach to the treatment of diabetic cardiomyopathy.

The present study contains several limitations. First, the STZ-induced type 1 diabetic mouse model was used, while in clinical practice, diabetic cardiomyopathy commonly occurs in patients with type 2 diabetes. However, current mouse models of type 2 diabetes manifest only left ventricular diastolic dysfunction, which is different from combined left ventricular systolic and diastolic dysfunction typically seen in clinical patients. Second, we did not explore the effects of Ang IV on AT_1_R, AT_2_R and MasR, nor the interaction between these receptors and AT_4_R, although a previous study did show that the therapeutic effect of Ang IV was blunted only by AT_4_R antagonist but not by AT_1_R, AT_2_R or MasR antagonists 14. Third, no gene knockout or transgenic mice were used, and the selectivity of pharmacological inhibitors is relative. Finally, as in most murine experiments, we used only male mice to avoid possible effect of estrogen on insulin resistance. Thus, there might be a sex difference if the same experiments were performed using female mice. Future studies are warranted to address these important issues.

## Conclusion

Ang IV treatment dose-dependently attenuated left ventricular dysfunction and remodeling in a mouse model of diabetic cardiomyopathy, and the mechanism involved stimulation of AT_4_R, suppression of FoxO1 nuclear translocation, and inhibition of FoxO1-mediated overactive autophagy. Thus, Ang IV and FoxO1 provide a promising therapeutic target for diabetic cardiomyopathy.

## Supplementary Material

Supplementary figures and tables, materials and methods.

## Figures and Tables

**Table 1:** Effects of Ang IV on left ventricular function and dimension in 5 groups of mice of the first part *in vivo* experiment

	NC	DM	Low-dose Ang IV	Medium-dose Ang IV	High-dose Ang IV
LVEF (%)	78.00 ± 2.90***	49.66 ± 3.94	59.27 ± 2.64^##^	61.73 ± 2.32*^#^	72.31 ± 1.98***
FS (%)	49.30 ± 2.58***	30.29 ± 3.56	34.24 ± 1.79^#^	36.73 ± 1.66	43.02 ± 2.02**
E/A	1.52 ± 0.07**	0.97 ± 0.14	1.12 ± 0.09^#^	1.31± 0.08	1.47 ± 0.05**
E'/A'	1.71 ± 0.11***	0.79± 0.14	1.02 ± 0.14	1.08 ± 0.11	1.33 ± 0.08*
LVEDD (mm)	2.78 ± 0.04**	3.45 ± 0.17	3.35 ± 0.12^#^	3.21 ± 0.10	2.85 ± 0.10**

Ang IV: Angiotensin IV; DM: diabetes mellitus; E/A: the ratio of the early to late diastolic mitral inflow velocities; E'/A': the ratio of the early to late diastolic mitral annular velocities; FS: fractional shortening; LVEF: left ventricular ejection fraction; LVEDD: left ventricular end-diastolic diameter; NC: normal control. *p < 0.05, **p < 0.01, ***p < 0.001 vs. DM group; ^#^*p* < 0.05, ^##^*p* < 0.01 vs. High-dose Ang IV group. n ≥ 8 per group.

**Table 2:** Effects of AT_4_R and FoxO1 on left ventricular function and dimension in 5 groups of mice of the second part *in vivo* experiment

	DM	Ang IV	Ang IV+ Divalinal	AS	Ang IV+AS
LVEF (%)	41.45 ± 3.50	63.34 ± 2.20***	43.79 ± 5.58^##^	60.54 ± 2.72***	67.76 ± 2.25***
FS (%)	25.72 ± 1.51	36.87 ± 2.09**	28.21 ± 2.62^#^	38.56 ± 1.52***	41.97 ± 2.93***
E/A	0.87 ± 0.06	1.36 ± 0.08***	1.00 ± 0.18^#^	1.27 ± 0.08**	1.45 ± 0.13**
E'/A'	0.59 ± 0.07	1.29 ± 0.13**	0.80 ± 0.15^#^	1.51 ± 0.10***	1.48 ± 0.13***
LVEDD (mm)	3.73 ± 0.12	3.19 ± 0.09**	3.67 ± 0.08^##^	3.20 ± 0.10**	3.08 ± 0.11***

Ang IV: Angiotensin IV; AS: FoxO1 inhibitor AS1842856; E/A: the ratio of the early to late diastolic mitral inflow velocities; E'/A': the ratio of the early to late diastolic mitral annular velocities; FS: fractional shortening; LVEF: left ventricular ejection fraction; LVEDD: left ventricular end-diastolic diameter. **p < 0.01, ***p < 0.001 vs. DM group; ^#^*p* < 0.05, ^##^*p* < 0.01 vs. Ang IV group. n ≥ 8 per group.

## Abbreviations

Ang II: angiotensin II
Ang IV: angiotensin IV
Ang-(1-7): angiotensin-(1-7)
AS: AS1842856
AT_1_R: Ang II type 1 receptor
AT_2_R: Ang II type 2 receptor
AT_4_R: Ang II type 4 receptor
Cl-caspase3: cleaved caspase 3
Col I: collagen I
Col III: collagen III
E/A: the ratio of the early to late diastolic mitral inflow velocities
E'/A': the ratio of the early to late diastolic mitral annular velocities
FBG: fasting blood glucose
FoxO1: forkhead box protein O1
FoxO1-OE: FoxO1 overexpression
FS: fractional shortening
HDL-C: high-density lipoprotein cholesterol
HG: high glucose
HO: high osmotic
LDL-C: low-density lipoprotein cholesterol
LVEDD: left ventricular end-diastolic diameter
LVEF: left ventricular ejection fraction
RAAS: rennin-angiotensin-aldosterone system
TC: total cholesterol
TG: triglycerides

## References

[1] Lehrke M, Marx N (2017). Diabetes mellitus and heart failure. Am J Med.

[2] Weng J, Zhou Z, Guo L, Zhu D, Ji L, Luo X (2018). Incidence of type 1 diabetes in China, 2010-13: population based study. BMJ.

[3] Li X, Wu Y, Zhao J, Wang H, Tan J, Yang M (2020). Distinct cardiac energy metabolism and oxidative stress adaptations between obese and non-obese type 2 diabetes mellitus. Theranostics.

[4] Evangelista I, Nuti R, Picchioni T, Dotta F, Palazzuoli A (2019). Molecular dysfunction and phenotypic derangement in diabetic cardiomyopathy. Int J Mol Sci.

[5] Hu L, Ding M, Tang D, Gao E, Li C, Wang K (2019). Targeting mitochondrial dynamics by regulating Mfn2 for therapeutic intervention in diabetic cardiomyopathy. Theranostics.

[6] Kenny HC, Abel ED (2019). Heart failure in type 2 diabetes mellitus. Circ Res.

[7] Feng Y, Xu W, Zhang W, Wang W, Liu T, Zhou X (2019). LncRNA DCRF regulates cardiomyocyte autophagy by targeting miR-551b-5p in diabetic cardiomyopathy. Theranostics.

[8] Hao P, Yang J, Liu Y, Zhang M, Zhang K, Gao F (2015). Combination of angiotensin-(1-7) with perindopril is better than single therapy in ameliorating diabetic cardiomyopathy. Sci Rep.

[9] Hao PP, Yang JM, Zhang MX, Zhang K, Chen YG, Zhang C (2015). Angiotensin-(1-7) treatment mitigates right ventricular fibrosis as a distinctive feature of diabetic cardiomyopathy. Am J Physiol Heart Circ Physiol.

[10] Yang JM, Dong M, Meng X, Zhao YX, Yang XY, Liu XL (2013). Angiotensin-(1-7) dose-dependently inhibits atherosclerotic lesion formation and enhances plaque stability by targeting vascular cells. Arterioscler Thromb Vasc Biol.

[11] Chansel D, Czekalski S, Vandermeersch S, Ruffet E, Fournie-Zaluski MC, Ardaillou R (1998). Characterization of angiotensin IV-degrading enzymes and receptors on rat mesangial cells. Am J Physiol.

[12] Smith AI, Turner AJ (2004). What's new in the renin-angiotensin system?. Cell Mol Life Sci.

[13] Yang H, Zeng XJ, Wang HX, Zhang LK, Dong XL, Guo S (2011). Angiotensin IV protects against angiotensin II-induced cardiac injury *via* AT4 receptor. Peptides.

[14] Park BM, Cha SA, Lee SH, Kim SH (2016). Angiotensin IV protects cardiac reperfusion injury by inhibiting apoptosis and inflammation *via* AT4R in rats. Peptides.

[15] Mei Y, Thompson MD, Cohen RA, Tong X (2015). Autophagy and oxidative stress in cardiovascular diseases. Biochim Biophys Acta.

[16] Ren J, Taegtmeyer H (2015). Too much or not enough of a good thing-the Janus faces of autophagy in cardiac fuel and protein homeostasis. J Mol Cell Cardiol.

[17] Porrello ER, Delbridge LM (2009). Cardiomyocyte autophagy is regulated by angiotensin II type 1 and type 2 receptors. Autophagy.

[18] Lin L, Liu X, Xu J, Weng L, Ren J, Ge J (2016). Mas receptor mediates cardioprotection of angiotensin-(1-7) against angiotensin II-induced cardiomyocyte autophagy and cardiac remodelling through inhibition of oxidative stress. J Cell Mol Med.

[19] Nakamura M, Sadoshima J (2020). Cardiomyopathy in obesity, insulin resistance and diabetes. J Physiol.

[20] Xing YQ, Li A, Yang Y, Li XX, Zhang LN, Guo HC (2018). The regulation of FoxO1 and its role in disease progression. Life Sci.

[21] van der Vos KE, Eliasson P, Proikas-Cezanne T, Vervoort SJ, van Boxtel R, Putker M (2012). Modulation of glutamine metabolism by the PI(3)K-PKB-FOXO network regulates autophagy. Nat Cell Biol.

[22] Iwai-Kanai E, Yuan H, Huang C, Sayen MR, Perry-Garza CN, Kim L (2008). A method to measure cardiac autophagic flux in vivo. Autophagy.

[23] Matsuzaki T, Alvarez-Garcia O, Mokuda S, Nagira K, Olmer M, Gamini R (2018). FoxO transcription factors modulate autophagy and proteoglycan 4 in cartilage homeostasis and osteoarthritis. Sci Transl Med.

[24] Jia G, Whaley-Connell A, Sowers JR (2018). Diabetic cardiomyopathy: a hyperglycaemia- and insulin-resistance-induced heart disease. Diabetologia.

[25] Dong B, Yu QT, Dai HY, Gao YY, Zhou ZL, Zhang L (2012). Angiotensin-converting enzyme-2 overexpression improves left ventricular remodeling and function in a rat model of diabetic cardiomyopathy. J Am Coll Cardiol.

[26] Zhao YX, Yin HQ, Yu QT, Qiao Y, Dai HY, Zhang MX (2010). ACE2 overexpression ameliorates left ventricular remodeling and dysfunction in a rat model of myocardial infarction. Hum Gene Ther.

[27] Kong J, Zhang K, Meng X, Zhang Y, Zhang C (2015). Dose-dependent bidirectional effect of angiotensin IV on abdominal aortic aneurysm *via* variable angiotensin receptor stimulation. Hypertension.

[28] Ma H, Guo R, Yu L, Zhang Y, Ren J (2011). Aldehyde dehydrogenase 2 (ALDH2) rescues myocardial ischaemia/reperfusion injury: role of autophagy paradox and toxic aldehyde. Eur Heart J.

[29] Mialet-Perez J, Vindis C (2017). Autophagy in health and disease: focus on the cardiovascular system. Essays Biochem.

[30] He C, Zhu H, Li H, Zou MH, Xie Z (2013). Dissociation of Bcl-2-Beclin1 complex by activated AMPK enhances cardiac autophagy and protects against cardiomyocyte apoptosis in diabetes. Diabetes.

[31] Tong M, Saito T, Zhai P, Oka SI, Mizushima W, Nakamura M (2019). Mitophagy is essential for maintaining cardiac function during high fat diet-induced diabetic cardiomyopathy. Circ Res.

[32] Yao Q, Ke ZQ, Guo S, Yang XS, Zhang FX, Liu XF (2018). Curcumin protects against diabetic cardiomyopathy by promoting autophagy and alleviating apoptosis. J Mol Cell Cardiol.

[33] Mellor KM, Varma U, Stapleton DI, Delbridge LM (2014). Cardiomyocyte glycophagy is regulated by insulin and exposure to high extracellular glucose. Am J Physiol Heart Circ Physiol.

[34] Zhao L, Zhang Q, Liang J, Li J, Tan X, Tang N (2019). Astrocyte elevated gene-1 induces autophagy in diabetic cardiomyopathy through upregulation of KLF4. J Cell Biochem.

[35] Zhao Y, Yang J, Liao W, Liu X, Zhang H, Wang S (2010). Cytosolic FoxO1 is essential for the induction of autophagy and tumour suppressor activity. Nat Cell Biol.

[36] Jash S, Puri V (2016). FoxO1-autophagy axis regulates lipid droplet growth *via* FSP27. Cell Cycle.

[37] Yan D, Cai Y, Luo J, Liu J, Li X, Ying F (2020). FOXO1 contributes to diabetic cardiomyopathy *via* inducing imbalanced oxidative metabolism in type 1 diabetes. J Cell Mol Med.

[38] Li W, Du M, Wang Q, Ma X, Wu L, Guo F (2017). FoxO1 promotes mitophagy in the podocytes of diabetic malenmice *via* the PINK1/Parkin pathway. Endocrinology.

[39] Auguste G, Gurha P, Lombardi R, Coarfa C, Willerson JT, Marian AJ (2018). Suppression of activated FoxO transcription factors in the heart prolongs survival in a mouse model of laminopathies. Circ Res.

