# Supplementary material for: Angiotensin IV attenuates diabetic cardiomyopathy via suppressing FoxO1-induced excessive autophagy, apoptosis and fibrosis
Source: Theranostics. 2021 Jul 25;11(18):8624–39. doi: 10.7150/thno.48561 (PMC8419053; doi:10.7150/thno.48561)
Supplement: Supplementary file 1 — Supplementary figures and tables, materials and methods. [file thnov11p8624s1.pdf]

Supplemental data

Angiotensin IV attenuates diabetic cardiomyopathy *via* suppressing FoxO1-induced excessive autophagy, apoptosis and fibrosis

Short title: *Ang IV attenuates diabetic cardiomyopathy*

By:

Meng Zhang[1], Wenhai Sui[1], Yanqiu Xing[2], Jing Cheng[3], Cheng Cheng[1], Fei Xue[1], Jie Zhang[1], Xiaohong Wang[1], Cheng Zhang[1*], Panpan Hao[1*], and Yun Zhang[1*]

From:

[1]The Key Laboratory of Cardiovascular Remodeling and Function Research, Chinese Ministry of Education, Chinese National Health Commission and Chinese Academy of Medical Sciences, The State and Shandong Province Joint Key Laboratory of Translational Cardiovascular Medicine, Department of Cardiology, Qilu Hospital, Cheeloo College of Medicine, Shandong University, Jinan 250012, Shandong, China.

[2]Department of Geriatrics, Qilu Hospital, Cheeloo College of Medicine, Shandong University, Key Laboratory of Cardiovascular Proteomics of Shandong Province, Jinan 250012, Shandong, China.

[3]Beijing Chaoyang Hospital, Capital Medical University, Beijing 100020, China.

*_**Correspondence:**_ Yun Zhang, MD, PhD, FACC, FESC, FASE, E-mail: zhangyun@sdu.edu.cn, or Panpan Hao, MD, PhD, E-mail: panda.how@sdu.edu.cn, or Cheng Zhang, MD, PhD, FACC, FESC, E-mail:

zhangc@sdu.edu.cn, Department of Cardiology, Qilu Hospital, Cheeloo College of Medicine, Shandong University, Jinan, Shandong, 250012, China.

Table S1. Effects of Ang IV on fasting serum lipid and glucose levels and blood pressure in 5 groups of mice of the first part *in vivo* experiment

	NC	DM	Low-dose Ang IV	Medium-dose Ang IV	High-dose Ang IV
TC (mmol/L)	3.18 ± 0.40	2.48 ± 0.33	2.41 ± 0.24	2.45 ± 0.20	2.38 ± 0.15
LDL-C (mmol/L)	2.14 ± 0.17	1.93 ± 0.14	1.68 ± 0.14	1.94 ± 0.13	1.71 ± 0.12
HDL-C (mmol/L)	0.77 ± 0.10	0.84 ± 0.10	0.57 ± 0.09	0.69 ± 0.12	0.60 ± 0.07
TG (mmol/L)	1.40 ± 0.22	1.19 ± 0.17	0.99 ± 0.09	1.18 ± 0.12	0.93 ± 0.09
GLU (mmol/L)	6.30 ± 1.05	19.42 ± 1.64***	21.63 ± 2.06***	23.23 ± 1.19***	24.21 ± 1.13***
SBP (mmHg)	116.4 ± 3.95	113.8 ± 3.76	115.1 ± 3.76	114.4 ± 3.22	115.2 ± 3.67
MBP (mmHg)	93.24 ± 3.26	94.57 ± 4.11	97.15 ± 4.95	96.02 ± 2.71	88.48 ± 4.06
DBP (mmHg)	84.58 ± 3.68	83.79 ± 3.71	77.64 ± 3.52	83.91 ± 5.90	79.64 ± 5.22

Ang IV: angiotensin IV; DBP: diastolic blood pressure; DM: diabetes mellitus; GLU: glucose; HDL-C: high-density lipoprotein cholesterol; LDL-C: low-density lipoprotein cholesterol; MBP: mean blood pressure; NC: normal control; SBP: systolic blood pressure; TC: total cholesterol; TG: triglycerides. ***$p < 0.001$ vs. the NC group. $n \geqslant 8$ per group.

Table S2. Effects of FoxO1 and AT_4R on fasting serum lipid and glucose levels and blood pressure in 5 groups of mice of the second part *in vivo* experiment

	DM	Ang IV	Ang IV+ Divalinal	AS	Ang IV+AS
TC (mmol/L)	2.30 ± 0.25	2.59 ± 0.20	2.29 ± 0.23	2.44 ± 0.14	2.55 ± 0.10
LDL-C (mmol/L)	1.69 ± 0.09	1.86 ± 0.14	1.87 ± 0.15	1.65 ± 0.09	1.84 ± 0.17
HDL-C (mmol/L)	0.80 ± 0.09	0.70 ± 0.06	0.73 ± 0.07	0.60 ± 0.05	0.68 ± 0.06
TG (mmol/L)	1.18 ± 0.10	1.23 ± 0.09	1.36 ± 0.18	1.28 ± 0.09	1.17 ± 0.12
GLU (mmol/L)	22.86 ± 1.12	23.46 ± 1.12	20.99 ± 1.18	22.64 ± 1.40	24.27 ± 1.08
SBP (mmHg)	112.9 ± 4.14	117.3 ± 3.87	115.4 ± 3.28	117.3 ± 3.81	113.0 ± 3.39
MBP (mmHg)	92.36 ± 4.23	90.11 ± 3.87	93.78 ± 4.81	90.21 ± 2.69	87.88 ± 4.02
DBP (mmHg)	79.40 ± 5.03	73.88 ± 5.81	80.80 ± 4.88	75.93 ± 4.90	71.80 ± 3.92

Ang IV: angiotensin IV; AS: AS1842856; DBP: diastolic blood pressure; DM: diabetes mellitus; GLU: glucose; HDL-C: high-density lipoprotein cholesterol; LDL-C: low-density lipoprotein cholesterol; MBP: mean blood pressure; SBP: systolic blood pressure; TC: total cholesterol; TG: triglycerides. $n \geqslant 8$ per group.

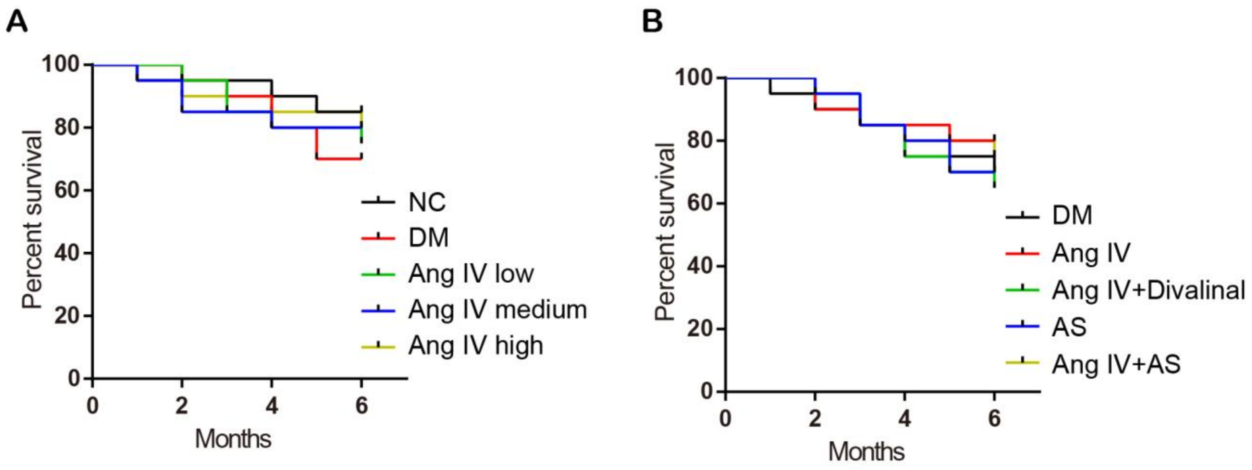

figure S1

Figure S1. Effects of Ang IV, AT_4R and FoxO1 on survival rate in 5 groups of mice, respectively. (A) The Kaplan-Meier survival curves in 5 groups of mice of the first part *in vivo* experiment. **(B)** The Kaplan-Meier survival curves in 5 groups of mice on the second part *in vivo* experiment.

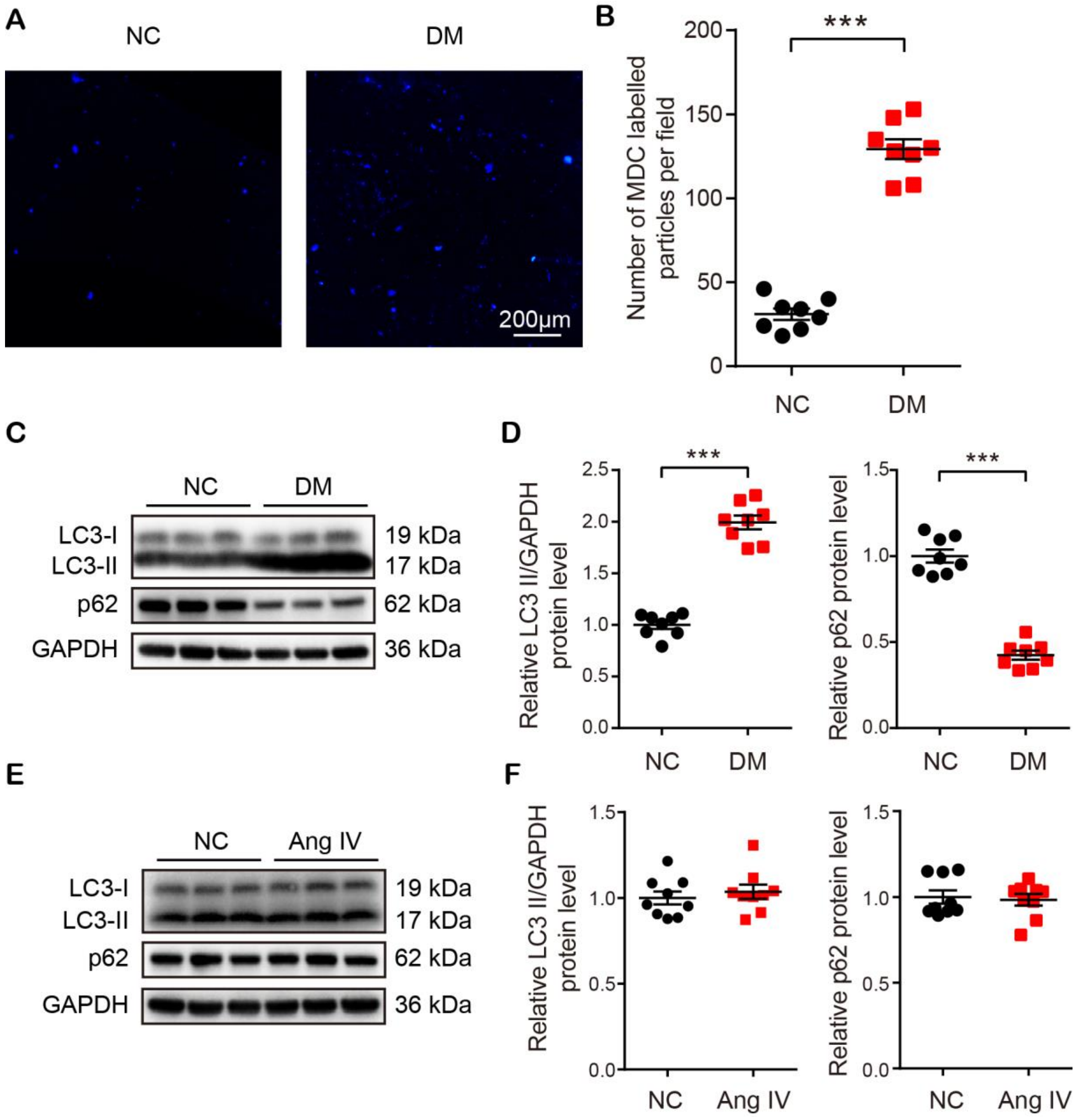

Figure S2. Effects of DM and Ang IV on myocardial autophagy in mice.

(A) Representative images of MDC labelled particles in the myocardium of NC and DM groups of mice. **(B)** Quantification of MDC labelled particles in the myocardium of NC and DM groups of mice. n=8 per group. **(C)** Representative Western blot images of LC3 and p62 in the NC and DM groups of mice. **(D)** Quantification of LC3-II and p62 expressions in the NC and DM groups of mice. n=8 per group. **(E)** Representative Western blot images of LC3 and p62 in the

myocardium of normal mice treated with vehicle or Ang IV. **(F)** Quantification of LC3-II and p62 expressions in NC and Ang IV groups of normal mice. n=9 per group. Ang IV: angiotensin IV; DM: diabetes mellitus; NC: normal control. ****p* < 0.001.

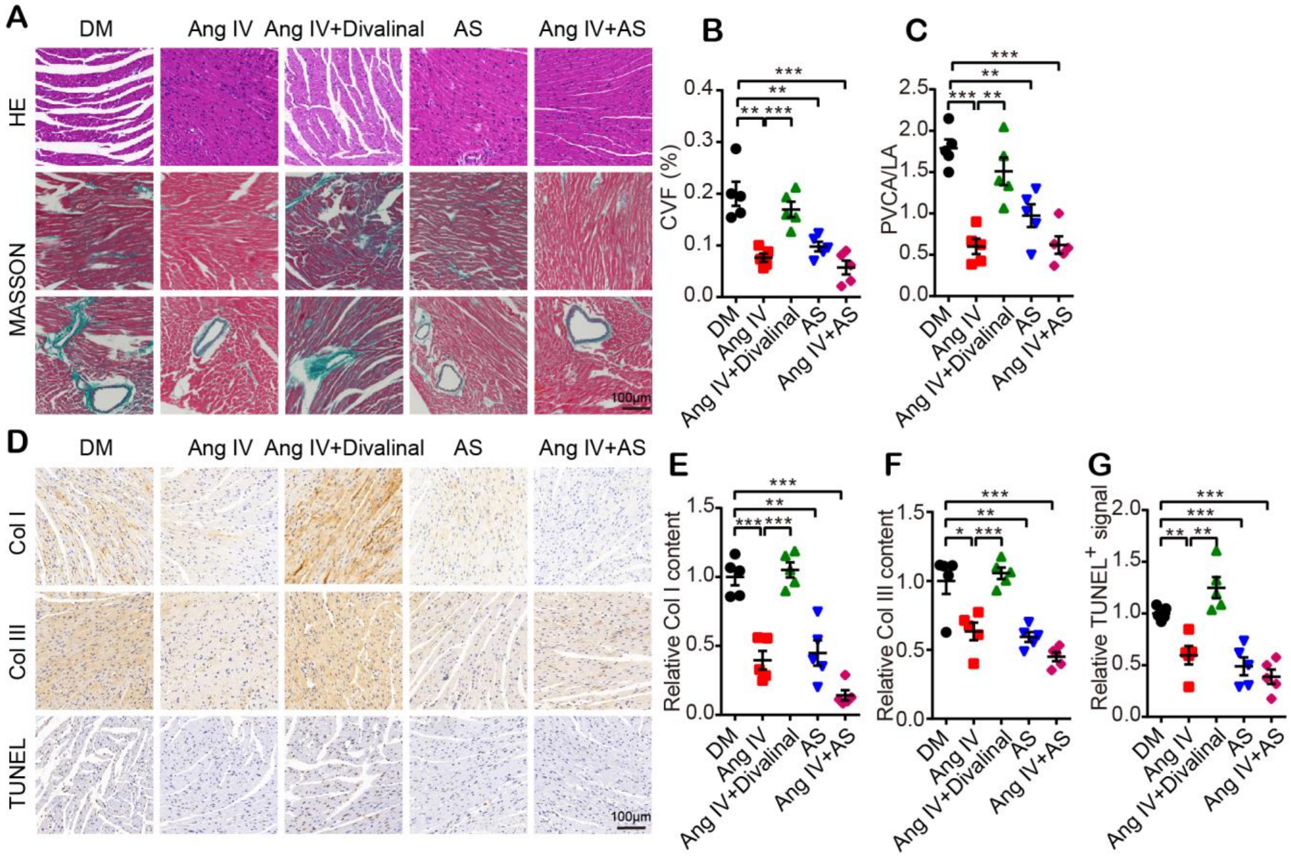

Figure S3. Effects of AT4R and FoxO1 on myocardial fibrosis and apoptosis in 5 groups of mice of the second part *in vivo* experiment. (A) Representative H&E and Masson's trichrome in 5 groups of mice. **(B-C)** Quantification of collagen volume fraction (CVF) and the ratio of perivascular collagen area to luminal area (PVCA/LA). **(D)** Representative immunohistochemical staining of Col I, Col III, and TUNEL in 5 groups of mice. **(E-G)** Quantification of immunohistochemical staining of Col I, Col III, and TUNEL staining. n=5 per group. Ang IV: angiotensin IV; AS: AS1842856; Col I:

collagen I; Col III: collagen III; DM: diabetes mellitus. *$p < 0.05$, **$p < 0.01$, and ***$p < 0.001$.

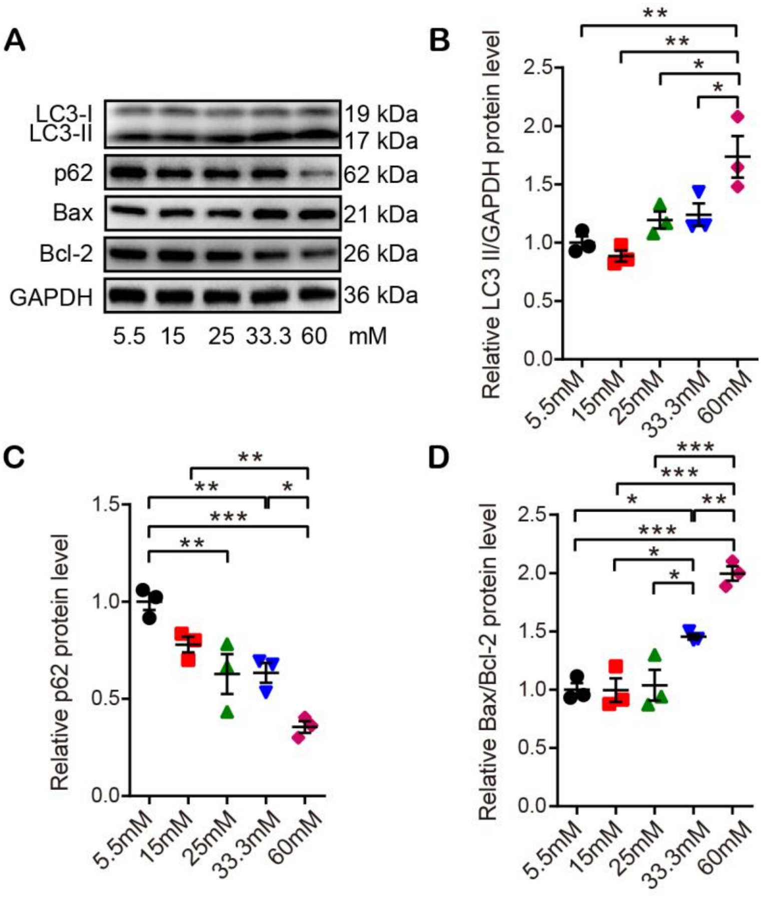

Figure S4. Effects of different concentrations of glucose on expressions of apoptosis- and autophagy-associated proteins in H9C2 cardiomyocytes. (A) Representative Western blot images of LC3, p62, Bax and Bcl-2 in 5 groups of cells. **(B-D)** Quantification of LC3-II level, p62 level and Bax/Bcl-2 ratio in 5 groups of cells. n=3 per group. *$p < 0.05$, **$p < 0.01$, and ***$p < 0.001$.

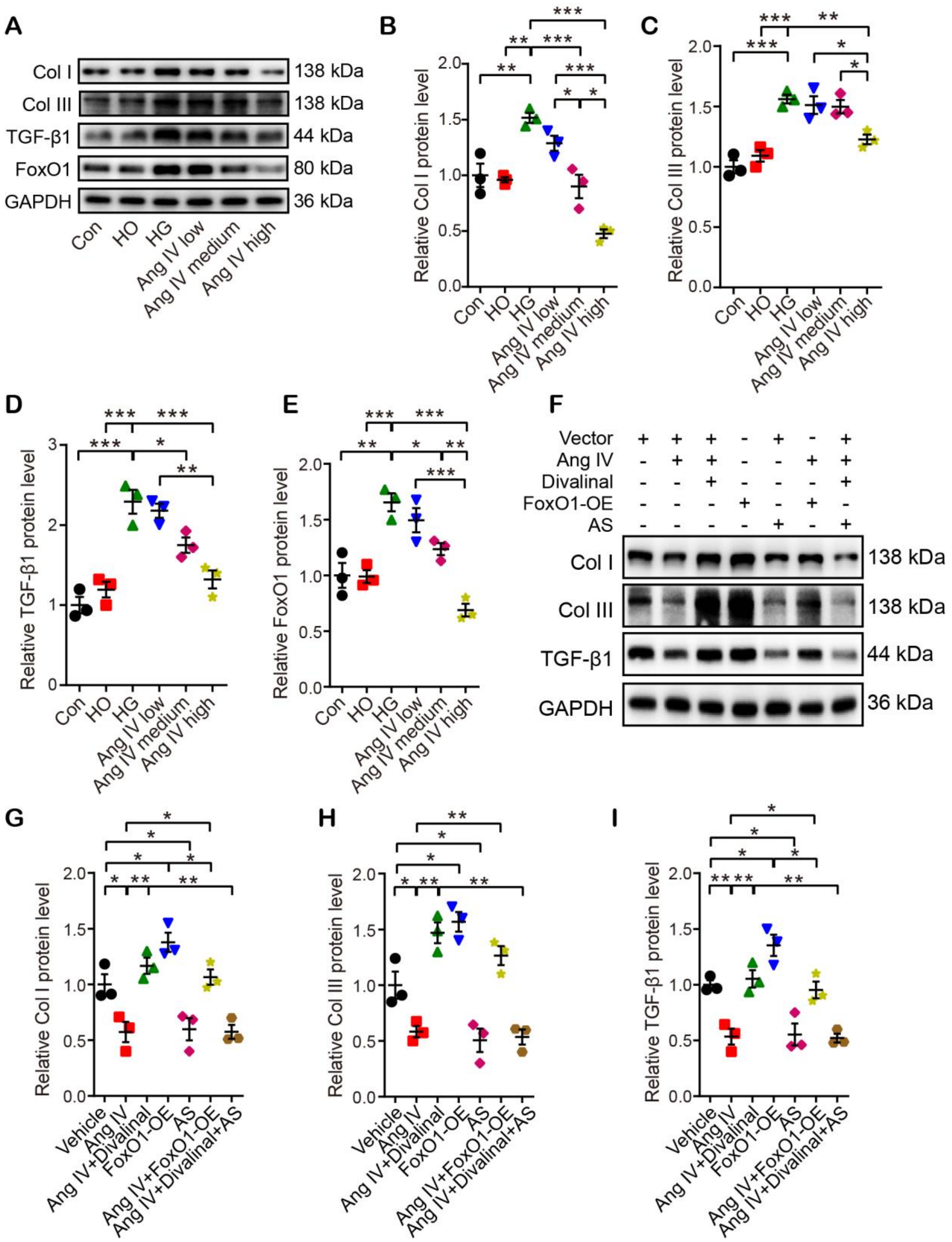

Figure S5. Effects of Ang IV, AT_4R and FoxO1 on the expressions of fibrosis-associated markers in cardiac fibroblasts. (A) Representative Western blot images of Col I, Col III, TGF-β1 and FoxO1 in 6 groups of cells.

(B-E) Quantification of Col I, Col III, TGF-β1 and FoxO1 expressions in 6 groups of cells. **(F)** Representative Western blot images of Col I, Col III and TGF-β1 in 7 groups of cells. **(G-I)** Quantification of Col I, Col III and TGF-β1 expressions in 7 groups of cells. n=3 per group. Ang IV: angiotensin IV; AS: AS1842856; Con: normal glucose control; Col I: collagen I; Col III: collagen III; FoxO1-OE: FoxO1 overexpression; HG: high glucose; HO: high osmotic control. *$p < 0.05$, **$p < 0.01$, and ***$p < 0.001$.

Materials and methods

Effects of DM and Ang IV on myocardial autophagy in mice

To examine the effect of DM on myocardial autophagy (Fig. S2A-2D), 24 eight-week-old male mice with C57BL/6J background were randomly divided into NC and DM groups (12 mice in each group). DM was induced in mice via intraperitoneal injection of STZ as described in the text. Mice in the NC group were injected with vehicle (0.1 mL of citrate buffer, pH 4.5) instead of STZ. After feeding with a normal chow for 24 weeks, chloroquine (Cq, MCE, HY-17589A, 10 mg/kg) was intraperitoneally injected into the NC and DM mice. Three hours after injection, monodansyl cadaverine (MDC, MCE, HY-D1027, 1.5 mg/kg) was intraperitoneally injected into mice. An hour after MDC injection, mice were euthanized and their hearts were harvested. Cryostat sections of the heart were obtained and proteins from the heart were extracted for Western blot analysis.

To clarify the effect of Ang IV on myocardial autophagy in normal mice (Fig. S2E-2F), 24 eight-week-old male mice with C57BL/6J background were randomly divided into NC and Ang IV groups (12 mice in each group). Saline or Ang IV (2.88 mg/kg/day) were respectively infused into mice via subcutaneous osmotic mini-pumps for 16 weeks. Then mice were euthanized and their hearts were harvested. Proteins were extracted for Western blot analysis.

Echocardiographic imaging

Transthoracic echocardiography was performed at the end of 24 weeks using

the Vevo2100 imaging system (VisualSonics, Toronto, Canada) under 2% isoflurane anesthesia on a heated platform. Left ventricular end-diastolic diameter (LVEDD), and left ventricular ejection fraction (LVEF) and fractional shortening (FS) were measured by M-mode echocardiography in the parasternal long-axis view. The early (E) and late (A) diastolic mitral flow velocities were measured by pulsed Doppler in the apical four-chamber view, and the ratio of E/A was calculated. The early (E') and late (A') diastolic mitral annular velocities were measured by tissue Doppler imaging in the apical four-chamber view, and the ratio of E'/A' was derived.

Blood pressure measurement

Blood pressure was measured by a noninvasive tail-cuff system (Softron BP-98A, Tokyo, Japan) in all mice that were trained first to adapt to the device to ensure reproducible measurements. Blood pressure was measured between 9:00 AM and 11:00 AM by the same operator and recorded as the mean of three consecutive measurements.

Biochemical assay

The serum levels of total cholesterol (TC), low-density lipoprotein cholesterol (LDL-C), high-density lipoprotein cholesterol (HDL-C) and triglycerides (TG) were measured in all mice by using a commercial kit (Roche, Mannheim, Germany). The serum level of fasting blood glucose (FBG) was measured using an Accu Chek glucose meter and the matched blood glucose strips (Roche, Mannheim, Germany).

Histological and immunochemical staining

Freshly excised hearts were fixed in 4% paraformaldehyde, paraffin-embedded, and sectioned into 4-μm thick slices. Hematoxylin and eosin (H&E) staining was performed to display cardiomyocyte morphology. Masson's trichrome staining was used to display collagen deposition. Sections were dewaxed, incubated overnight at 4 °C with corresponding primary antibody against collagen I (Col I; 1:100 dilution), collagen III (Col III; 1:50), microtubule-associated protein 1 light chain 3 β (LC3; 1:100 dilution), Beclin1 (1:100) or p62 (1:100; all Abcam, Cambridge, MA), and subsequently incubated with corresponding secondary antibody for 30 min at 37 °C. Then sections were stained with diaminobenzidine and hematoxylin.

LC3 was stained with an autophagy kit (KGAF004, KeyGEN, China) following the manufacturer's instructions. In brief, cardiomyocytes were stimulated with indicated agents for 12 h, and chloroquine diphosphate (30 μM) was added to the medium and incubated for the next 12 h. Thereafter, cells were fixed with 4% paraformaldehyde and stained with LC3 antibody and FITC-labeled secondary antibody. All histological images were analyzed with the Image-Pro Plus 6.0 software.

Transmission electron microscopy

Myocardial tissues were isolated from mice of all groups (n=3 each), fixed with 2.5% glutaraldehyde, post-fixed with 1% osmium tetroxide, dehydrated through a graded ethanol series, and embedded in epoxy resin. Ultra-thin

sections (90 nm thick) were double-stained with uranyl acetate and lead citrate, and then the images were captured using a transmission electron microscope (H-7000FA, Hitachi, Tokyo, Japan).

Detection and quantitation of apoptosis

Apoptotic cells in tissue sections were detected with a TUNEL detection kit (Roche, Germany) according to the manufacturer's instructions. In brief, slides were de-waxed and incubated with TdT and dUTP mixture for 2 h at 37 °C. Then converter-POD was added to tissues followed by diaminobenzidine and hematoxylin staining.

Microarray protocol

Myocardial tissues were isolated from mice of NC, DM, and DM+high-dose Ang IV groups (n=4) for microarray. Total RNA was extracted using Trizol reagent (Life Technologies, Carlsbad, CA) and purified with an RNeasy mini kit (Qiagen, Valencia, CA). Biotinylated cDNA was prepared according to the standard Affymetrix protocol from 250 ng total RNA. Following labeling, fragmented cDNA was hybridized for 16 h at 45 °C using the Clariom™ S Assay (Affymetrix, Santa Clara, CA). GeneChips were washed and stained in the Affymetrix Fluidics Station 450. All arrays were scanned by using Affymetrix® GeneChip Command Console (AGCC) which was installed in GeneChip® Scanner 3000 7G**.**

Affymetrix GeneChip standard hybridization quality control was bioB<bioC<bioD<cre; neg<pos. The raw data were normalized by the

Transcriptome Analysis Console software (version: 4.0.1) with Robust Multichip Analysis (RMA) algorithm using Affymetrix default analysis settings and global scaling as a normalization method. The values presented were $\log_2$ RMA signal intensity and the microarray data are publicly available at NCBI Gene Expression Omnibus (GEO) under accession number GSE157331.

In microarrays, we used the limma R package (version: 3.36.5) based on moderated F-statistic to filter the differentially expressed genes (DEGs). Empirical Bayes moderation was used to correct the p values. The Benjamini-Hochberg method was used for multiple test correction (false discovery rate was used to adjust the p values for multiple comparisons). The threshold set for up- and down-regulated genes was fold change > 2.0, p-value < 0.05 and false discovery rate < 0.05.

Hierarchical clustering was performed based on differentially expressed mRNAs using an R package heatmap (version: 1.0.12). To clarify the biological functions of the genes and the involved signaling pathways, we annotated each gene based on the Gene Ontology (GO) and Kyoto Encyclopedia of Genes and Genomes (KEGG) databases. Enrichment calculations were performed using Fisher's exact test. We further conducted GO and pathway enrichment analysis of the target genes. The specific principle was to carry out annotation mapping of DEGs in GO and KEGG database entries, calculate the number of the target genes in each GO and pathway entry, and then use the hypergeometric test for statistics. The GO and KEGG entries that were

significantly enriched in the DEGs were selected. After the calculated p-value was corrected by multiple hypothesis tests, the p-value 0.05 was taken as the threshold, and the GO and KEGG terms meeting this condition were defined as significant enrichment.

Western blot analysis

Proteins of murine hearts and cardiomyocyte extracts were separated by SDS-PAGE and transferred to polyvinylidene fluoride membranes for incubation with primary antibodies against Col I (1:1000 dilution), Col III (1:500), TGF-β1 (1:1000), Bax (1:1000), Bcl-2 (1:1000), LC3 (1:4000), Beclin1 (1:1000), GAPDH (1:1000; all Abcam), SQSTM1/p62 (1:1000), FoxO1 (1:1000), pFoxO1 (1:1000) and cleaved caspase 3 (Cl-caspase3; 1:1000) (all CST, Danvers, MA) overnight at 4 °C and appropriate secondary antibodies (1:5000; Proteintech, Wuhan, China) for 1 h at room temperature. Protein levels were normalized to that of GAPDH.

Cytoplasmic and nuclear extraction

The separation experiment of cytoplasmic and nuclear extraction was performed by using Minute™ Cytoplasmic & Nuclear Extraction Kits (Invent, SC-003), following the standard protocol. Briefly, cells were harvested in suspension by low-speed centrifugation (500 *g* for 3 min), and the supernatant was aspirated completely. Cytoplasmic extraction buffer was added to cell pellets, and the tube was vortexed vigorously for 15 s, incubated on ice for 5 min, and then centrifuged for 5 min at top speed (14,000~16,000 *g*) in a

microcentrifuge at 4 °C. The supernatant (cytosol fraction) was transferred into a fresh pre-chilled 1.5 mL tube. Nuclear extraction buffer was added to the pellets, and the tube was vortexed vigorously for 15 s, and then incubated on ice for one min. The vortexing and incubation were repeated for 4 times. Then, the nuclear extract was immediately transferred to a pre-chilled filter cartridge with a collection tube and centrifuged at top speed in a microcentrifuge for 30 s. The filter cartridge was discarded, and the nuclear extract was stored at -80 °C until use.

Primary culture of cardiac fibroblasts

Neonatal mice were anesthetized with isoflurane (0.5%) in a gas chamber and cleaned with 70% ethanol. Hearts were removed from mice, cut into small pieces and put into spinning flasks (50~70 rpm) with D-Hank's digestive solution (containing 0.0125% collagenase II) at 4 °C overnight. Then the supernatant was discarded and a new D-Hank's digestive solution (containing 0.0125% pancreatin, without EDTA) was added to cover the tissues. The flasks were incubated in the water bath at 37 °C at a low spinning speed for 2 min followed by pipetting up and down gently. The supernatant was collected carefully and the same volume of Dulbecco's modified Eagle's medium (DMEM; Gibco BRL, Gaithersburg, MD) containing 10% FBS was added to stop the digestion. The new D-Hank's digestive solution (containing 0.0125% pancreatin, without EDTA) was added to cover the rest tissues, and the procedures were repeated until all tissues were digested. The pooled cells

were centrifuged for 5 min at 1,500 *g*, and the supernatant was discarded. Cell pellets were resuspended using DMEM and centrifuged for 5 min at 1,500 *g*. The supernatant was discarded, and the cells were plated in dishes in DMEM and incubated at 37 °C in 5% CO_2. After cell culture for 1.5-2 h, cells adhered to the dish were cardiac fibroblasts.
